# Regionally enriched rare deleterious exonic variants in the UK and Ireland

**DOI:** 10.1038/s41467-024-51604-2

**Published:** 2024-10-02

**Authors:** Mihail Halachev, Viktoria-Eleni Gountouna, Alison Meynert, Gannie Tzoneva, Alan R. Shuldiner, Colin A. Semple, James F. Wilson

**Affiliations:** 1 MRC Human Genetics Unit, Institute of Genetics and Cancer, University of Edinburgh, Edinburgh, UK; 2 Regeneron Genetics Center, Tarrytown, NY USA; 3 Centre for Global Health Research, Usher Institute, University of Edinburgh, Edinburgh, UK; 4 Centre for Genomic and Experimental Medicine, Institute of Genetics and Cancer, University of Edinburgh, Edinburgh, UK

**Keywords:** Rare variants, High-throughput screening

## Abstract

It is unclear how patterns of regional genetic differentiation in the UK and Ireland might impact the protein-coding fraction of the genome. We exploit UK Biobank (UKB) and Viking Genes whole exome sequencing data to study regional genetic differentiation across the UK and Ireland in protein coding genes, encompassing 44,696 unrelated individuals from 20 regions of origin. We demonstrate substantial exonic differentiation among Shetlanders, Orcadians, individuals with full or partial Ashkenazi Jewish ancestry and in several mainland regions (particularly north and south Wales, southeast Scotland and Ireland). With stringent filtering criteria, we find 67 regionally enriched (≥5-fold) variants likely to have adverse biomedical consequences in homozygous individuals. Here, we show that regional genetic variation across the UK and Ireland should be considered in the design of genetic studies and may inform effective genetic screening and counselling.

## Abstract (web summary)

The genetic variants present in humans often reflect the geographical origins of their ancestors. This study shows that people from different UK regions tend to carry different rare variants in their exomes, with potential biomedical consequences.

## Introduction

Geographically diverse human populations often exhibit distinct profiles of genomic variation. This was first established based on mitochondrial DNA^1^ and Y chromosome haplotyping^2,3^ and the advent and mass adoption of next-generation sequencing technologies soon made clearer the true breadth and complexity of this phenomenon. For example, the 1000 Genomes Project conducted whole-genome sequencing (WGS) and analysis of 2504 individuals from 26 populations in Africa, East Asia, Europe, South Asia and the Americas and found while the most common variants are not limited by geography, the vast majority of variants (86%) are constrained to continental groups and rarer variants are typically restricted to closely related populations^4^. Such differential signal persists even at smaller geographical distances and is evident even when only a subset of the full genomic variation is investigated. Analysing genome-wide single nucleotide polymorphism (SNP) genotyping data of 2039 individuals from rural areas within the UK (and with grandparents within the same areas), Leslie et al.^5^ showed remarkable concordance between genetic and geographic clustering of samples across the country. Further differentiation was reported by Gilbert at al.^6^ based on genome-wide SNP genotyping data analysis of 2544 individuals from five different cohorts of regional English, Welsh, Scottish, Manx, or Irish ancestry.

Isolated populations can show more extreme divergence due to strong genetic drift. The European Ashkenazi Jewish (AJ) population has long been regarded as a genetic isolate showing clear evidence for genetic drift arising from population bottlenecks, endogamy, as well as complex patterns of admixture and selection at particular loci^7^. We have previously found strong genetic drift in the isolated Shetland population in northern Scotland, relative to the more cosmopolitan mainland Scottish population^8^. Many of the ultra-rare exonic variants found to be enriched in Shetland are predicted to impact gene function and may affect biomedical traits^9^, consistent with similar enrichments observed in other geographically isolated populations^10–13^. The Shetland population’s demographic history reflects the substantial physical barriers to immigration historically, and it is thought that over the last 200–300 years many regions of the UK may have experienced limited migration^14,15^, preserving regional genetic clusters that appear to reflect more ancient histories of those regions^5,16^. However, it is unclear whether these patterns of regional differentiation have any relevance to health and disease.

UK Biobank (UKB) is a large-scale biomedical database providing medical and genetic data to accredited researchers from half a million volunteer participants with the aim of enabling new scientific discoveries and improving public health^17^. A UMAP analysis of the genome-wide SNP data of 488,377 UKB participants confirmed the previously observed regional genetic stratification in the UKB, showing clear clustering based on self-reported ethnic background, as well as north-south and east-west gradients^18^. This large-scale and richly annotated dataset has already provided valuable insights into human health and disease. A plethora of association studies revealed numerous genotype-phenotype associations for common/complex diseases, with some focusing on the effect of common variants^19–21^, while others investigated the contribution of rare variation^22,23^.

In this work, using a subset of the whole-exome sequencing (WES) data available for UKB participants^24^ focused on self-identified “White British” individuals born outside large metropolitan areas, combined with a unique collection of WES data from the Northern Isles of Scotland, we reveal three main insights. First, we demonstrate the two Northern Isles populations (Shetland and Orkney) are remarkably genetically distinct from mainland populations, and despite their geographical proximity, from each other. Second, we show that the previously observed UK regional separation based on genome-wide genotyping data can be broadly recapitulated using genetic variation from the protein-coding part of the genome only. Lastly, our analyses based on stringent filtering criteria identify a variety of exonic variants predicted to have detrimental health effects, which although generally extremely rare, are nonetheless enriched in particular UK regions.

## Results

Based on the regional availability of participants with WES data in UKB we classified samples into 16 geographical regions of origin (Methods). These regions contain individuals who were born within the corresponding region, but outside large metropolitan areas, who self-identify as “White British” and who exhibit very similar genetic ancestry based on a principal components analysis of the UKB whole-genome SNP array genotypes. There are two exceptions: the London region which contains individuals born in a 10 mile radius area around the geographical centre of London (i.e., a cosmopolitan control) and the Irish region for which we selected individuals who self-identify as “Irish” and were born in either Northern Ireland or the Republic of Ireland. We also included UKB participants with Ashkenazi Jewish (AJ) heritage, which we split into two groups (full and part AJ) based on their genomic information (Methods). Lastly, we added WES data from two cohorts in the Viking Genes programme^9,25^, from the relatively isolated archipelagos of Shetland and Orkney (the Northern Isles of Scotland), for which the sequencing and variant calling procedures were identical to those utilized for UKB WES data generation, for a total of 20 regions and 44,696 unrelated individuals (Supplementary Fig. 1).

### Individuals with Ashkenazi Jewish heritage in UKB

According to the 2021 UK census, more than quarter of a million respondents answered “Jewish” to the voluntary question on religion. Recent studies have found evidence of participation of such individuals in the UKB project, including a study based on identity-by-descent (IBD) analysis of the 500k UKB participants^26^ and a recent analysis of European haplotype sharing in UKB SNP genotyping data^27^. An independent clustering analysis based on UKB whole-genome SNP array genotypes also revealed a distinct group of UK individuals, which based on their genetic data and UKB lifestyle questionnaire answers are likely to be of Jewish ancestry. Our further analysis of these individuals using the UKB WES data indicated that this group is enriched for some known pathogenic variants causing disorders with higher prevalence in Ashkenazi Jewish (AJ) individuals, including a frameshift variant in the *HEXA* gene causing Tay-Sachs disease (rs387906309, ~50x enrichment in our Jewish ancestry group compared to Central London) and a missense variant in the *GBA* gene causing Gaucher disease, Type I (rs76763715, ~13x enrichment).

Our WES-based Multi-Dimensional Scaling (MDS) analysis revealed the existence of two main clusters within this group (Supplementary Fig. 2A). Our hypothesis that these two groups of individuals in the UKB dataset are distinct from each other is supported by two lines of evidence: (a) an MDS analysis based on known biallelic SNPs shows clear separation between these two groups when compared to a control group consisting of London individuals (Supplementary Fig. 2B); (b) a higher total number of runs of homozygosity (ROH)^28^ and a higher overall proportion of each individual’s genome was observed in ROH for one of these two groups compared to the other, demonstrating lesser amount of admixture (Supplementary Fig. 2C). These observations, combined with the fact that currently the vast majority (95%^29^) of British Jews are Ashkenazi, lead us to believe that these two groups presumably consist of individuals with full AJ (e.g. with 3 or more AJ grandparents) or part AJ (e.g., 2 or fewer AJ grandparents or with other Jewish heritage) heritage. Hereafter we refer to these two groups as full AJ and part AJ for brevity, noting that we cannot rule out the possibility that some Jewish individuals with different heritage (e.g. Sephardi, Mizrahi, Yemenite, Iraqi, Iranian or Georgian Jewish) may also be present in them. We included full AJ (1004 unrelated individuals) and part AJ (657 unrelated Individuals) in the following analyses as representative groups of a well-established human isolate population which are at different stages of admixture with other populations, and serving as archetypal groups enriched for variants that are rare elsewhere.

### Enrichment of shared ultra-rare SNP alleles in the Northern Isles

To check for any potential batch/regional effects in sample collection, storage, manipulation and bioinformatics processing, we computed the overall variation load for each of the 20 regions, after performing extensive QC filtering (Methods) of the variants discovered by the UKB alignment and variant calling OQFE protocol. We found that except individuals with AJ heritage, the samples from the remaining 18 regions exhibit virtually identical variant loads with medians of 31,885 exonic SNPs and 823 INDELs (short insertions or deletions) per person (Supplementary Table 1). To investigate the slight total SNP variant enrichment observed for individuals with AJ heritage compared to their non-AJ counterparts (~1% for full AJ and ~0.5% for part AJ), we further split the 20 regional variant datasets to “ultra-rare”, containing variants which have not been observed in any individual in the gnomAD genome dataset (v3.1.1, *n* = 76,156), and “known”, for variants found in any gnomAD subpopulation^30^ with passing variant quality. Compared to the 18 non-AJ regions, which have ultra-rare and known variant allele loads comparable to each other (Supplementary Table 1), the two AJ groups exhibit a lower number of ultra-rare variants and higher number of known variants (i.e. previously observed in gnomAD), with the latter group driving up the overall AJ variant load. These observations for the variant load in the two AJ groups can be explained by the relatively high genomic homogeneity in such individuals and the inclusion of variation data from 1736 AJ participants in the gnomAD dataset (2.3% of all 76,156 individuals).

We also observed significant enrichment of shared ultra-rare SNP alleles in the Northern Isles, such that two-thirds of the ultra-rare variants found in Shetland are shared by two or more unrelated individuals from this region and more than half of the ultra-rare variants in Orkney are shared among individuals located there; in contrast, for example, only one-fifth of ultra-rare SNP alleles were observed to be shared among individuals within the London region. This finding confirms our previous result, which has been attributed to founder effects and increased genetic drift in the isolated Shetland population^8^. We note that the amount of shared ultra-rare variants in Orkney may be underestimated, due to the presence of 23 Orcadian individuals in the gnomAD dataset (via their inclusion in the 1000 Genomes project^4^), thus potentially reducing the overall number of ultra-rare variants found in Orkney.

### Rare exonic variation is associated with birthplace

**Fig. 1:**
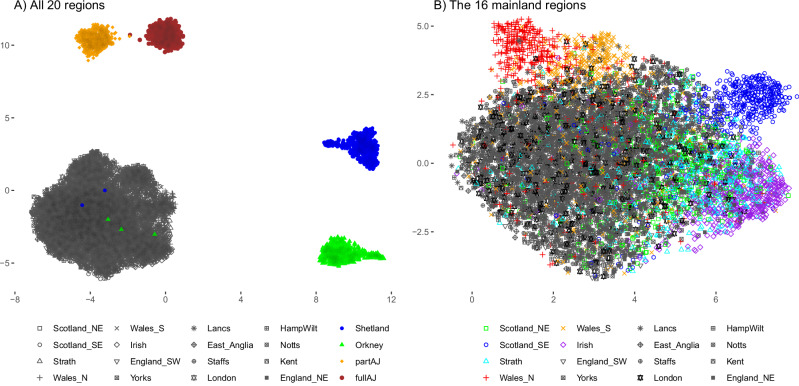
Distinctions among regional populations based upon UMAP projections of rare exonic variation. The UMAP projections are computed on the top 20 MDS dimensions discovered based on biallelic, non-singleton and linkage-disequilibrium (LD) pruned known SNPs with MAF < 5% in the considered unrelated individuals. (**A**) UMAP analysis of all 20 groups in our study illustrating the clear genetic distinction of full AJ, part AJ, Shetland and Orkney individuals from each other and from mainland regions. Despite the careful curation of the genealogical records of the Northern Isles participants, some carry a significant proportion of UK mainland heritage; (**B**) UMAP analysis focusing on the 16 mainland regions, recapitulating previously known distinctions among Welsh, English, Scottish and Irish regions.

Recent research based on genome-wide genotyping arrays has demonstrated a striking association between genomic variation and place of birth for individuals in the UK and the Republic of Ireland^5,6^. To assess if this geographical distinction can be recapitulated based on exonic data only, we assembled a dataset of 10,001 unrelated individuals from the UKB and the Northern Isles (492 Shetlandic, 509 Orcadian and 500 randomly chosen individuals from the remaining 18 groups). Performing MDS/UMAP analyses based upon rare (MAF < 5%) exonic SNP variation in the joint dataset of the 20 regions (Methods) using the top 20 MDS dimensions reveals a clear distinction of full AJ, part AJ, Shetland and Orkney populations from each other and from mainland regions (Fig. 1A, Supplementary Fig. 3A). Focusing on the 16 mainland UK and Ireland regions similarly based upon rare (MAF < 5%) exonic SNP variation in their joint dataset, distinctions among Welsh, English, Scottish and Irish exomes are evident, consistent with previous studies based on genome-wide genotyping arrays^5,6^ (Fig. 1B, Supplementary Fig. 3B). In addition, our analysis reveals an additional differentiation between North and South Welsh individuals, and suggests some level of separation exhibited by individuals born in South East Scotland (Fig. 1B), both of which have been previously observed^5^. Our choice of using rare SNP variants (MAF < 5%) is driven by the empirical observation that it is the most suitable threshold since it best recapitulates the previously published results (Supplementary Fig. 4).

We also computed the pair-wise *F*_*ST*_ distances (Methods) based upon biallelic, non-singleton, linkage-disequilibrium (LD)-pruned known SNPs with MAF < 5% across the 20 geographical regions (the same set of variants used for the MDS/UMAP analyses above) as another measure of the exonic distance between the regions (Supplementary Table 2). The results further highlighted the clear exonic distinctiveness of the AJ and Northern Isles populations to the 16 mainland regions (Supplementary Fig. 5), suggesting that the individuals from Shetland (mean *F*_*ST*_ = 0.00091) and Orkney (mean *F*_*ST*_ = 0.00083) represent a degree of genetic divergence from the mainland regions in their exomes that is comparable to the divergence of the part AJ (mean *F*_*ST*_ = 0.00090). In accord with the MDS analysis, Irish, Welsh and mainland Scottish regions show elevated mean *F*_*ST*_ distances to each other and to the English (0.00024, 0.00015, 0.00011, respectively), compared to comparisons within England or mainland Scotland (mean *F*_*ST*_ = 0.00005, 0.00004, respectively). The unrooted phylogenetic tree (Supplementary Fig. 6) we built based upon the pair-wise *F*_*ST*_ distances reiterates the Welsh-English-Scottish-Irish differentiation revealed by our MDS/UMAP analysis.

### Identification of regionally enriched deleterious variants

**Fig. 2:**
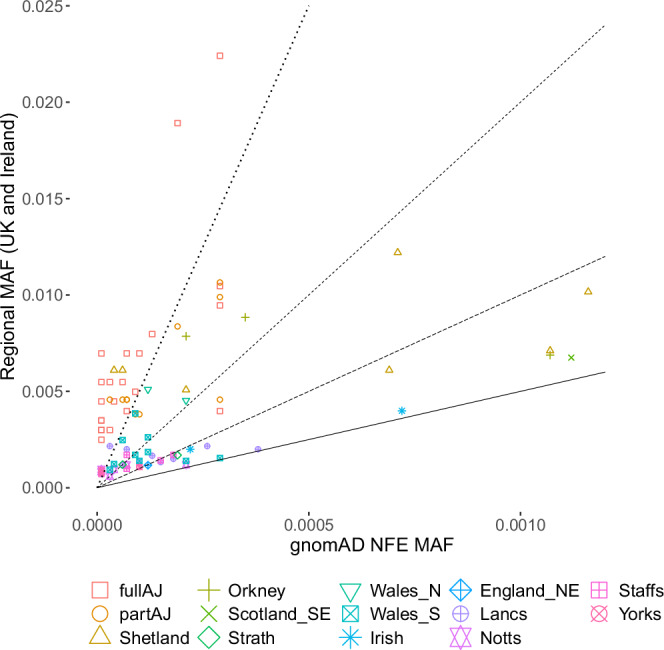
Regionally enriched deleterious variants discovered in the UKB regions of the UK and Ireland. Each of the 67 discovered variants is represented as a point with the frequency at which it is found in gnomAD NFE individuals (*x*-axis) and its regional frequency (*y*-axis). Note that, for visual clarity, the two axes are on different scales. To facilitate variant enrichment interpretation, added are four guide lines representing variant regional MAF enrichment of 5 times (solid line), 10 times, 20 times and 50 times (dotted lines) compared to gnomAD NFE. Precise enrichment information per each variant is available in the subsequent tables.

Based on the observed regional stratification in UKB, we sought evidence for the presence of potentially deleterious exonic variants enriched in particular geographical regions. We conservatively restricted our analysis to variants predicted to affect the coding potential of canonical transcripts, causing stop codon gain, start codon loss, splice donor/acceptor site loss, and frameshifts, as well as missense and splice region variants confidently predicted to be deleterious (CADD score ≥ 30). From the variants identified in these classes we then defined as enriched those found at a regional frequency at least 5 times higher than the frequency observed in gnomAD NFE and attaining statistical significance (Methods). Overall, we discovered at least one enriched and potentially deleterious variant in 14 of the considered 20 UKB regions, summing up to 67 unique variants. These variants are: (i) enriched in one or more of the UKB regions compared to NFE in gnomAD, (ii) predicted to be functional, (iii) implicated in a monogenic disorder and (iv) reported in ClinVar^31^ to be pathogenic/likely pathogenic (Methods). The vast majority (95%) of the discovered variants are previously known, but extremely rare variants, with 90% of these having gnomAD MAF_NFE_ < 0.0004 (Fig. 2).

We would like to clarify at this point that all the reported enriched variants are implicated in recessive disorders, i.e. individuals carrying the variant in a heterozygous state (referred to as “carriers”, with only one variant copy of the gene) are not affected; in order to be affected individuals must be homozygous. Thus, given that UKB and Viking Genes participants are generally healthy, it is not surprising that all 67 reported variants discovered in our dataset are observed only in a heterozygous state. In the subsequent regional sections, we provide more information on the disorders associated with each of these variants. We also include an estimate (HOM_ALT_) of the number of individuals in each region, who may be expected to develop a disorder by inheriting two variant copies of the gene from their parents, based on the regional carrier frequency and various mating patterns.

### Analysis of the reference AJ group within UKB is instructive

Our analysis revealed 24 enriched and potentially deleterious exonic variants in UKB participants with AJ descent, with 10 of these variants being shared by full and part AJ, while the remaining 14 are seen exclusively in full AJ (Supplementary Table 3). Most of the identified variants are correlated with health conditions previously reported to be significantly enriched in individuals with Jewish origins^32^ – nine are predominantly AJ diseases, three are mostly found in Sephardi-Mizrahi Jewish and two are common in all Jewish groups (Supplementary Table 3). In addition, there is a higher incidence of various types of Retinitis pigmentosa among individuals with Jewish heritage^32^, as well as increased risk of developing breast and ovarian cancer among AJ women^33^. The rediscovery of these variants in our UKB analysis supports the effectiveness and accuracy of our approach for identifying deleterious variants enriched in UKB regions.

### Enriched and deleterious rare exonic variants in Scotland

We discovered nine enriched (with *p*-values reported in Supplementary Table 4) and potentially deleterious variants in the regions of Scotland considered in our analysis (Table 1). Four of these variants are specific to the Shetland Islands, with one specific variant found in each of the Orkney, Strathclyde and South East Scotland regions. Two variants were also found to be shared across regions of Scotland: a variant associated with Usher syndrome found to be enriched in Shetland and Strathclyde and another associated with Bardet-Biedl syndrome appearing as enriched in both of the Northern Isles populations. For each of the identified variants we also computed a range for the predicted regional number of individuals homozygous for the variant (HOM_ALT_ range, Table 1), with the lower bound based on the assumption of random mating of a region’s individuals with the whole of the UK and Ireland (with MAF_AVE_ representing the average variant MAF across the 20 regions in our study) and the upper bound based assuming random mating within the region only.

**Table 1:** Enriched and potentially deleterious variants in samples from Scotland

ClinVar Variant Allele ID	Gene	Condition	Region	MAF_REG_	Enrichment (vs MAF_NFE_)	MAF_AVE_	HOM_ALT_ range
33889	*CLCN1*	Congenital myotonia	Shetland	0.0061	138x	0.00008	[0,1]
21837	*ADGRV1*	Usher syndrome	Shetland Strathclyde	0.0061 0.0012	104x 21x	0.00020	[0,1] [1,3]
23042	*RDH5*	Fundus albipunctatus	Orkney	0.0088	25x	0.00035	[0,2]
23943	*PPT1*	Neuronal ceroid lipofuscinosis 1	Shetland	0.0122	17x	0.00067	[0,3]
21382	*FANCF*	Fanconi anaemia	Strathclyde	0.0017	8.8x	0.00026	[1,7]
20604	*AIPL1*	Leber congenital amaurosis	Shetland	0.0061	8.8x	0.00038	[0,1]
20006	*ABCG8*	Sitosterolaemia 1	Shetland	0.0102	8.8x	0.00124	[0,2]
16367	*BBS10*	Bardet-Biedl syndrome	Shetland Orkney	0.0071 0.0069	6.6x 6.4x	0.00128	[0,1] [0,1]
176561	*LOXHD1*	Nonsyndromic hearing loss and deafness	Scotland SE	0.0068	6.0x	0.00133	[13,65]

MAF_REG_: regional MAF of the variant.

MAF_NFE_: MAF of the variant in Non-Finnish European individuals in gnomAD.

MAF_AVE_: average MAF of the variant in the dataset of 10,001 unrelated individuals from 20 UK and Ireland regions.

HOM_ALT_ range: predicted range of the regional number of individuals homozygous for the variant.

### Enriched and deleterious rare exonic variants in Wales

We identified nine enriched (Supplementary Table 4) and potentially deleterious variants in the Welsh groups in UKB, eight of which were specific to South Wales and one shared with individuals born in North Wales (Table 2). The lack of north Wales specific variants is likely to be explained by the almost four-fold smaller sample size for unrelated North Welsh (*n* = 883) individuals in our study compared to their Southern counterparts (*n* = 3239). Furthermore, it is possible that not all eight South Wales variants are truly specific to this region; some may be shared with the neighbouring English regions (e.g. Gloucestershire, Herefordshire, Shropshire and Cheshire), which were not included in our study due to the insufficient number of unrelated UKB individuals in these regions with WES data available.

**Table 2:** Enriched and potentially deleterious variants in samples from Wales

ClinVar Variant Allele ID	Gene	Condition	Region	MAF_REG_	Enrichment (vs MAF_NFE_)	MAF_AVE_	HOM_ALT_ range
20826	*SLC7A9*	Cystinuria	Wales S	0.0039	44x	0.00048	[4,37]
133510	*CHEK2*	Hereditary cancer-predisposing syndrome	Wales N Wales S	0.0051 0.0026	43x 22x	0.00051	[2,18] [3,16]
71108	*NPHS1*	Finnish congenital nephrotic syndrome	Wales S	0.0025	42x	0.00031	[2,15]
16142	*AGL*	Glycogen Storage Disease Type III	Wales S	0.0009	32x	0.00015	[0,2]
815895	*SMARCAL1*	Schimke immuno-osseous dysplasia	Wales S	0.0012	28x	0.00015	[0,3]
203537	*GAMT*	Deficiency of guanidinoacetate methyltransferase	Wales S	0.0017	19x	0.00029	[1,7]
27983	*SPR*	Dystonia	Wales S	0.0019	16x	0.00025	[1,9]
105746	*CEP290*	Leber congenital amaurosis	Wales S	0.0014	14x	0.00029	[1,5]
414917	*MME*	Charcot-Marie-Tooth disease, axonal, type 2 T	Wales S	0.0015	5.2x	0.00044	[2,5]

MAF_REG_: regional MAF of the variant.

MAF_NFE_: MAF of the variant in Non-Finnish European individuals in gnomAD.

MAF_AVE_: average MAF of the variant in the dataset of 10,001 unrelated individuals from 20 UK and Ireland regions.

HOM_ALT_ range: predicted range of the regional number of individuals homozygous for the variant.

### Enriched and deleterious rare exonic variants in England

Our analysis of the WES data from individuals born in the ten English regions discovered 22 enriched (Supplementary Table 4) and potentially deleterious variants (Table 3). Apart from a single variant found to be enriched in the North East England region (in the *PNP* gene), all of the remaining 21 variants were identified in four neighbouring regions: Lancashire, Staffordshire, Nottinghamshire and Yorkshire. In addition to variants specific to each of these regions, we also identified three variants (in the *COL7A1*, *F11* and *COL4A4* genes) as shared between two of these regions and one variant (*ALMS1* gene) shared by individuals born in Lancashire, Staffordshire and Nottinghamshire.

**Table 3:** Enriched and potentially deleterious variants in samples from England

ClinVar Variant Allele ID	Gene	Condition	Region	MAF_REG_	Enrichment (vs MAF_NFE_)	MAF_AVE_	HOM_ALT_ range
226048	*PEX6*	Zellweger syndrome	Lancs	0.0022	73x	0.00030	[1,7]
32480	*COL7A1*	Dystrophic epidermolysis bullosa	Notts Lancs	0.0010 0.0008	65x 57x	0.00028	[0,1] [0,1]
186978	*G6PC*	Glycogen storage disease	Yorks	0.0009	62x	0.00011	[1,4]
185684	*CLPB*	3-Methylglutaconic aciduria with cataracts, neurologic involvement, and neutropenia	Notts	0.0008	57x	0.00018	[0,1]
359454	*COL7A1*	Dystrophic epidermolysis bullosa	Yorks	0.0008	52x	0.00023	[1,3]
431680	*PDE6A*	Retinitis pigmentosa	Staffs	0.0007	48x	0.00016	[0,0]
19010	*ALMS1*	Alstrom syndrome	Lancs Staffs Notts	0.0020 0.0017 0.0012	27x 23x 16x	0.00064	[2,6] [2,3] [1,1]
611902	*PEPD*	Prolidase deficiency	Notts	0.0010	22x	0.00017	[0,1]
249211	*FIG4*	Amyotrophic lateral sclerosis	Notts	0.0006	20x	0.00018	[0,0]
190033	*DRAM2*	Retinal Dystrophy	Lancs	0.0012	20x	0.00031	[1,2]
100244	*DYSF*	Limb-girdle muscular dystrophy	Staffs	0.0010	14x	0.00026	[0,1]
100251	*DYSF*	Limb-girdle muscular dystrophy	Staffs	0.0010	14x	0.00015	[0,1]
26935	*F11*	Hereditary factor XI deficiency disease	Lancs Yorks	0.0013 0.0011	13x 10x	0.00039	[1,3] [2,7]
33396	*CHRNE*	Myasthenic syndrome	Lancs	0.0017	13x	0.00045	[1,4]
240612	*DNAI1*	Primary ciliary dyskinesia	Staffs	0.0011	11x	0.00021	[0,1]
29030	*PNP*	Purine-nucleoside phosphorylase deficiency	England NE	0.0012	10x	0.00026	[1,4]
443176	*COL4A4*	Alport syndrome	Staffs Lancs	0.0014 0.0013	9.6x 9.0x	0.00048	[1,2] [1,3]
203474	*PNPO*	Pyridoxal phosphate-responsive seizures	Staffs	0.0017	9.6x	0.00038	[1,3]
16237	*MARVELD2*	Deafness, autosomal recessive 49	Lancs	0.0015	8.5x	0.00043	[1,3]
272434	*SLC7A9*	Cystinuria	Lancs	0.0022	8.2x	0.00077	[3,7]
489962	*TECTA*	Nonsyndromic hearing loss and deafness	Notts	0.0012	5.8x	0.00034	[0,1]
76544	*DNAH5*	Primary Ciliary Dyskinesia	Lancs	0.0020	5.2x	0.00064	[2,6]

MAF_REG_: regional MAF of the variant.

MAF_NFE_: MAF of the variant in Non-Finnish European individuals in gnomAD.

MAF_AVE_: average MAF of the variant in the dataset of 10,001 unrelated individuals from 20 UK and Ireland regions.

HOM_ALT_ range: predicted range of the regional number of individuals homozygous for the variant.

### Enriched and deleterious rare exonic variants in Ireland

The analysis of the 2005 unrelated UKB individuals who self-identify as Irish and were born in either Northern Ireland or the Republic of Ireland resulted in identification of two enriched (Supplementary Table 4) and potentially deleterious variants (Table 4).

**Table 4:** Enriched and potentially deleterious variants in Irish individuals born on the island of Ireland

ClinVar Variant Allele ID	Gene	Condition	Region	MAF_REG_	Enrichment (vs MAF_NFE_)	MAF_AVE_	HOM_ALT_ range
401365	*SPG7*	Hereditary spastic paraplegia	Ireland	0.0020	9.0x	0.00027	[4,28]
31343	*FMO3*	Trimethylaminuria	Ireland	0.0040	5.5x	0.00095	[26,110]

MAF_REG_: regional MAF of the variant.

MAF_NFE_: MAF of the variant in Non-Finnish European individuals in gnomAD.

MAF_AVE_: average MAF of the variant in the dataset of 10,001 unrelated individuals from 20 UK and Ireland regions.

HOM_ALT_ range: predicted range of the regional number of individuals homozygous for the variant.

One reason for the relatively smaller number of enriched variants found in Ireland compared to other mainland UKB regions may be the different sample selection criteria - in contrast to our requirement for individuals in England, Scotland and Wales to exhibit very similar genetic ancestry based on a principal components analysis of the UKB whole-genome SNP array genotypes, the Irish participants were selected only based on self-identification as Irish and being born in Northern Ireland or the Republic of Ireland (Methods). As a result, it is possible that our sample of Irish individuals contains some with non-Irish ancestry, e.g. in the process of selecting Irish individuals we have identified and excluded six participants with AJ heritage. Another factor might be the relatively low number of Irish participants with available WES data. The analysed 2005 unrelated individuals represent the whole population of Ireland (about 7 million), thus inhibiting identification of potential within-Ireland differentiating signal(s).

### Cross-regional enriched and deleterious rare exonic variants

A deleterious variant causing a frameshift in the *OBSL1* gene (chr2:219568063:G > GT, c.1273dup, p.T425fs, rs762334954) was found to be regionally enriched in the Northern Isles of Scotland (Orkney and Shetland) and puzzlingly, in geographically distant Wales. However, upon closer examination the variant also appears to be measurably enriched in other UKB regions as well, but failing to meet our stringent enrichment criteria there (Supplementary Table 5). This variant has been previously reported to be associated with the 3-M syndrome^34^, an extremely rare autosomal recessive primordial growth disorder, characterised by distinct facial features, radiological abnormalities, normal intelligence and final adult height in the range of 115 – 150 cm. The exact prevalence of this disorder remains unclear, with around 200 reported cases world-wide as of 2012 since the first published report in 1975, but predicted to have increased substantially with the greater awareness of the disorder and increased availability of genetic testing^35^. To estimate the practical impact of the elevated frequency of the *OBSL1* variant, we considered its effect in each UKB region separately. The variant is predicted to exhibit regional genetic prevalence of individuals homozygous for it (computed as MAF_REG_^2^) of 1/16 k (~1500 times higher than gnomAD NFE individuals) in Orkney, 1/39 k (~600 times higher) in Shetland, 1/49k (~500 times higher) in North Wales and 1/518 k (~45 times higher) in South Wales. Assuming random mating within regions, it is expected there will be 1.4, 0.6, 1.4 and 5.2 homozygous individuals affected by the condition in the Orkney, Shetland, North Wales and South Wales regions, respectively. Given the mean MAF = 0.000467 in the remaining UKB regions, a genetic prevalence of 1/4.6 m (~5 times higher than NFE) can be expected assuming random mating, which translates to 10.7 individuals affected by the condition across these regions. Overall, due to the regionally elevated frequency of the *OBSL1* variant, we estimate that up to 19 individuals across the UK and Ireland could be affected by 3-M syndrome due to being homozygous for this variant.

### Comparison of regional population genetic metrics

Many factors could underlie the observed patterns of rare exonic variation across the 20 regions in our study. In previous work, we evaluated the roles played by founder effects, genetic drift and relaxation of purifying selection in shaping the isolated Shetland genome^8^. While founder effects appear to play a role in the more isolated populations in our study (e.g., Shetland, Orkney, full AJ), given the small amount of shared ultra-rare exonic variants per individual in other groups (Supplementary Table 1) it is unlikely that this is a major force driving the observed regional differentiation for the remaining regions. In this section, we provide a comparison of the 20 regions based on the estimates of several metrics designed to capture the effects various forces have on shaping the regional genetic landscapes. The data these analyses are based on have some important constraints, including the general UKB participation bias, the fact that WES data is only a small subset of whole-genome variation and is derived from the protein-coding regions which are known to be generally more intolerant to variation compared to the other parts of the human genome, the exclusion in our analyses of individuals born in large metropolitan areas and the lack of a more suitable reference dataset for individuals with AJ heritage. Therefore, we note that our results cannot be considered as absolute estimates of these population metrics, but are only to be used as means for comparing the 20 regions in our study.

### Regional variant frequency fluctuation

In previous section, we focused our analyses on a set of variants present in the gnomAD dataset^30^, with MAF in Non-Finnish European individuals less than 1% (MAF_NFE_ < 1%), which were found to be significantly enriched in one or more of the UKB regions. Here, we perform similar analysis on a subset of more common variants with 1% ≤ MAF_NFE_ < 5%, with each individual in our study carrying on average about 1100 such variants (Supplementary Table 1). While the former set is more informative for investigating monogenic disorders, the latter may contain variation relevant to complex polygenic traits and due to its larger size could provide more robust estimates of the regional variant fluctuations.

**Fig. 3:**
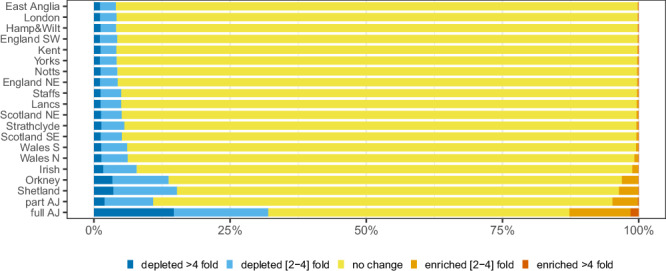
Estimate of regional variant frequency fluctuation. The numbers for each region represent the amount the SNPs in each category as a proportion of all regional SNPs found in Non-Finnish European (NFE) individuals with 1% ≤ MAF_NFE_ < 5%. The regions in the plot are sorted based on the total proportion of enrichment.

Our results (Fig. 3) show a clear correlation between the proportions of variants showing a significant regional frequency fluctuation compared to the general European population and the observed degree of distinctiveness from the relatively homogenous English regions (Fig. 1 and Figs. S3, 5, 6). We speculate that the observed proportions of regionally enriched and depleted variants are mainly driven by genetic drift. Compared to the relatively small amount of regionally enriched variants in the ten English, three mainland Scottish and two Welsh regions (from 0.19% for East Anglia to 0.77% for North Wales, mean = 0.34%, sd = 0.15%, Supplementary Table 6), which as well as regional variants will likely represent variants enriched at nation-wide level compared to Europe, the remaining regions—Irish (1.18%), Orkney (3.06%), Shetland (3.62%), part AJ (4.82%) and full AJ (12.7%) - exhibit a much higher proportion of regionally enriched variants, which corresponds well with their levels of geographical/cultural isolation.

Finally, all variants in a population are subject to genetic drift, including common variants. However, it has been shown that the variant frequency fluctuation in a population is inversely correlated with the initial variant frequency^36^. Thus, variants common in Non-Finnish Europeans (MAF_NFE_ > 5%) are expected to exhibit less informative regional fluctuations compared to those depicted in Fig. 3 (1% ≤ MAF_NFE_ < 5%). Furthermore, based on their relatively high frequency and the fact that exonic variants with MAF > 5% are mostly synonymous^37^, common variants are considered to be less likely to have strong detrimental impacts on human health. For these reasons, such variants are outside of the scope of the current work.

### Regional nucleotide diversity

One important factor which affects the strength of genetic drift and the regional variant frequencies is the past and present effective population size (*N*_*e*_). While a rigorous analysis of the regional historic and contemporary effective population sizes is outside of the scope of this work, for each region we have computed its nucleotide diversity (π = 4*N*_*e*_µ, where the mutation rate µ is generally similar across human populations). We use the estimated nucleotide diversity as a proxy for the effective population sizes potentially affecting the observed variant frequency fluctuations in the 20 regions.

Our nucleotide diversity analysis is based on 44,108 SNP variants with MAF > 5% in the dataset of 10,001 unrelated individuals from 20 regions which are also present in the full gnomAD dataset (Methods). Their presence in a public dataset indicates they are less likely to be sequencing and/or variant calling artefacts in our data, while their relatively high frequency (MAF > 5%) suggests they are less likely to have a functional impact and therefore alleviates the potential conflating signal from various forms of selection, which may affect our nucleotide diversity estimates.

**Fig. 4:**
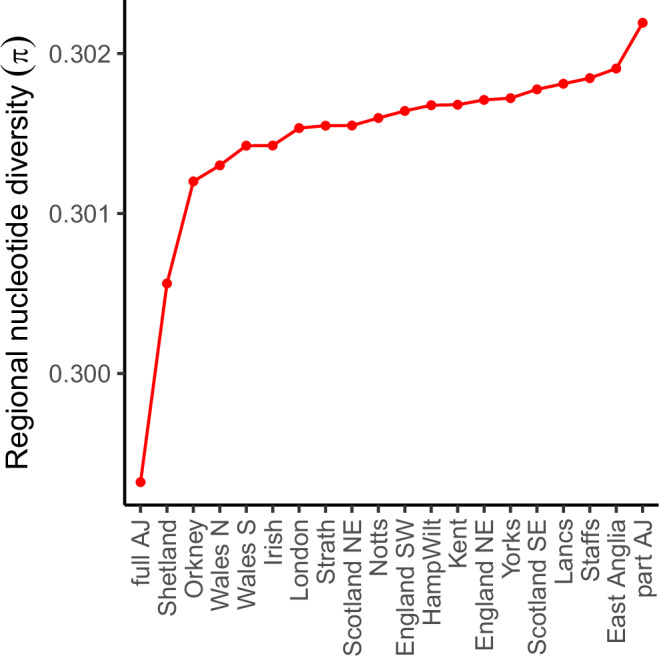
Estimate of regional nucleotide diversity. Lower π value implies smaller effective population size (*N*_*e*_). The regional π estimates are computed based on known SNPs found with MAF > 5% in our 20 region dataset.

Our results (Fig. 4, Supplementary Table 7) show that individuals with full AJ heritage exhibit the lowest nucleotide diversity followed by individuals from the other two isolated regions, Shetland and Orkney, the two Welsh regions and the Irish. The observed higher nucleotide diversity for the Northern Isles compared to the full AJ can be explained by the fact that although the population of each of the two archipelagos is an order of magnitude smaller than the predicted number of AJ individuals living in the UK, the nucleotide diversity in the AJ is strongly affected by their medieval bottleneck (estimated at *N* ~ 350)^38^, which was about an order of magnitude smaller than those in the Northern Isles^39^. The modern day Northern Isles genetic landscape was also significantly affected by the Scots-Norse admixture event^16,40,41^. While the remaining English and Scottish regions appear to have roughly similar nucleotide diversity, the individuals with part AJ heritage exhibit the highest nucleotide diversity in our dataset, due to their recent admixture with the remainder of the UK population.

### Regional strength of purifying selection

Another important factor having an effect on variation in protein-coding exonic regions is selection—variants improving Darwinian fitness increase their frequency via positive selection, while those with detrimental effects are removed by purifying selection. It has been shown previously that isolated populations, due to their smaller effective population size, exhibit weaker purifying selection^8,39^. Here, we investigate and compare the 20 regions based on the regional estimates of the strength of purifying selection.

Our estimate of the strength of the purifying selection is based on SNPs found in each regional cohort of 500 unrelated individuals with MAF ≤ 1% and not reported in the full gnomAD dataset, further split to LOF (including start lost, stop gained and splice acceptor/donor site) and synonymous variants. To account for the possibility that some of these ultra-rare variants may be sequencing/variant calling artefacts, we compare the 20 regions based on the mean number of LOF variants corrected by the mean number of synonymous variants discovered in an individual. By choosing variants not present in gnomAD, we focus our analysis on some potentially recent local variants (present in the UK and Ireland, but not reported or ultra-rare elsewhere) and by imposing the regional MAF threshold, we enrich for LOF variants that are likely functional (based on their predicted effect and their rarity in our data) and therefore subject to purifying selection. Our metric is similar to and inspired by the *SVxy* metric^39^, which cannot be directly applied in our context.

**Fig. 5:**
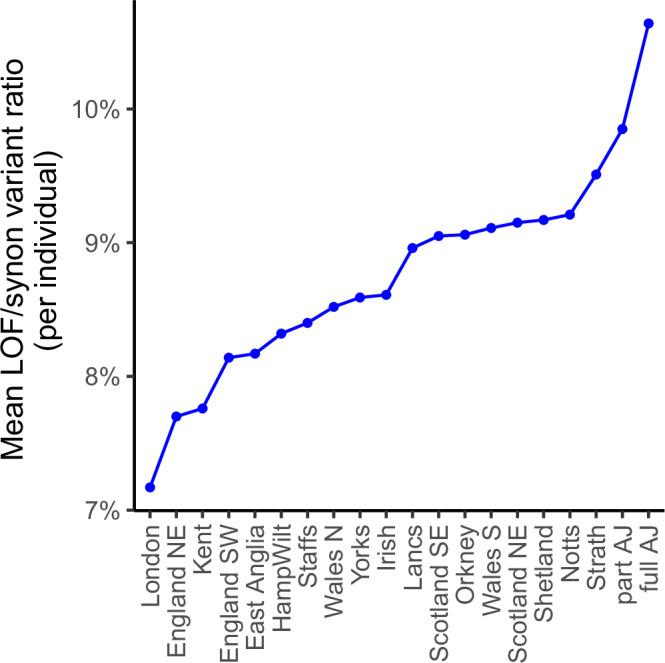
Estimate of regional strength of purifying selection. Lower LOF/synon ratio implies greater strength of purifying selection. The regional LOF/synon ratio estimates are computed based on variants with regional MAF ≤ 1% and not reported in the full gnomAD dataset. LOF: Loss of function variants, synon: synonymous variants.

Our results (Fig. 5) suggest that purifying selection is strongest in the cosmopolitan London region and weakest in the isolated full AJ group, with all Scottish regions, Nottinghamshire, Lancashire and South Wales exhibiting a somewhat weaker strength of purifying selection compared to the remaining regions. Individuals from Strathclyde have the highest LOF/synonymous ratio among non-AJ regions, however closer examination reveals that this is due to a disproportionate decrease in the mean number of the synonymous variants compared to the LOF variants discovered in this group (Supplementary Table 8). We note that while it can be expected that the regional strength of purifying selection will have a minimal impact on our reported set of 67 regionally enriched variants, due to their recessive nature, further research based on deeper and wider regional datasets may be informative in evaluating the effect of purifying selection in the dominant context.

### Cross-regional gene flow

The last factor with an impact on regional stratification which we considered is gene flow, which in our context translates to inter-regional mating and/or migration. To evaluate its effects, we used the previously computed pair-wise *F*_*ST*_ distance matrix converted to a similarity matrix by taking the reciprocals of the original values as an input to the R package qgraph^42^ (v 1.9.8) and generating the spring layout using the Fruchterman-Reingold algorithm^43^. In the resulting network, the distance between nodes is expected to correspond well to the absolute edge weight between those nodes and the colour saturation and the width of the edges corresponds to the absolute weight and scale relative to the strongest weight in the graph.

**Fig. 6:**
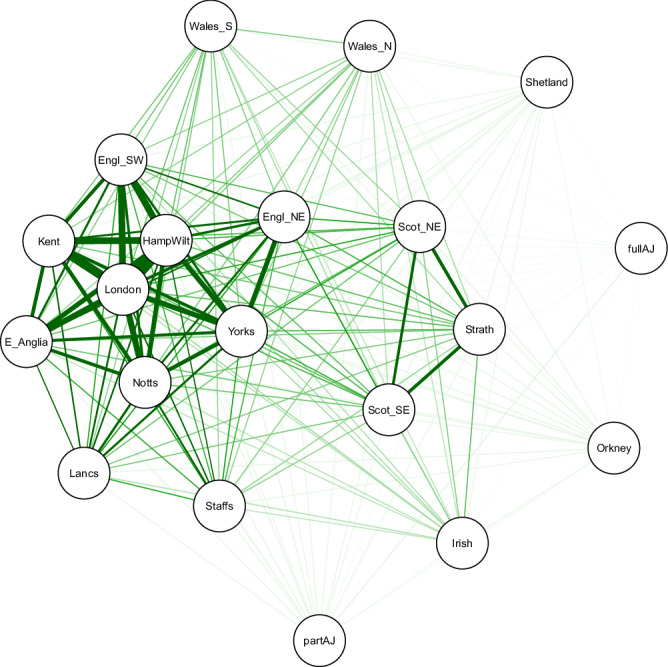
Estimate of cross-regional gene flow among the 20 regions. The cross-regional gene-flow estimate is computed using the calculated pair-wise *F*_*ST*_ distances among the regions using non-singleton known SNPs with MAF < 5% in our chosen set of 10,001 unrelated individuals (same set of variants used for the UMAP analysis presented in Fig. 1).

In addition to confirming the status of the Northern Isles and AJ as distanced to the mainland regions, our results (Fig. 6) provide several interesting observations. For example, while there is a signal of higher gene flow between the three mainland Scottish regions compared to the rest of the regions, no such observation can be made regarding the two Welsh regions—the gene flow between them appears to be on the same scale as with the remaining mainland regions, which potentially explains the clear separation of the two in our UMAP analysis (Fig. 1). Furthermore, while the ten English regions seem to form a loose cluster, there is a tighter hub-and-spokes cluster of seven English regions with London in the centre, which does not include the North East England, Lancashire and Staffordshire regions. Lastly, there is no evident signal of regional preference in the recent admixture of the part AJ group.

### Biomedical implications of the regionally enriched deleterious variation

The relatively high genomic homogeneity in individuals with Ashkenazi Jewish heritage has been well established and targeted genetic screens for variants implicated in various monogenic disorders have been adopted in Jewish populations world-wide. Enabled by the large-scale WES data in UK Biobank, we have demonstrated the existence of analogous deleterious variant enrichment within various geographical regions of the UK and Ireland (Tables 1–4). Our results highlight some single variants with large impact, for example the *FMO3* and *SPG7* gene variants in Irish individuals, the *SLC7A9* variant in South Wales, the *LOXHD1* variant in South East Scotland and the *CHEK2* variant in Welsh individuals. They also provide a disorder-centric view, for example Cystinuria with between 7 and 44 individuals predicted to be homozygous for the enriched causative variants, Primary ciliary dyskinesia with 2–7 individuals, Glycogen storage disease with 1–6 individuals, Leber congenital amaurosis with 1–6 individuals, and Dystrophic epidermolysis bullosa with 1–5 individuals. Additionally, based on our data, some regional aggregates may be estimated, for example between 30 and 138 Irish individuals predicted to be homozygous for the regionally enriched deleterious variants, 14–99 South Wales homozygous individuals, 13–65 South East Scotland individuals, 13–42 Lancashire individuals, etc. It should be noted that the reported numbers of predicted individuals homozygous for the regionally enriched deleterious variants are a clear underestimate of the number of individuals potentially affected by the corresponding condition due to the genetic and locus heterogeneity of the disorders, which is not taken into consideration in our calculations.

Given the non-negligible total number of individuals predicted to be affected by the reported regionally enriched variation, it appears reasonable to assume that this fact would have been ascertained through clinical means. However, given the variety of the implicated disorders, their general rarity and the potential aggregation at national level, we believe that our regionally/disorder-based approach provides unique insights and complements the existing awareness. Additionally, recent reports suggest that even for some well-studied conditions the clinical ascertainment may be suboptimal. For example, a large study focusing on pathogenic familial hypercholesterolaemia (FH) variants found that by employing targeted sequencing, almost half of the carriers were not previously known to their health provider and received a new diagnosis of FH^44^. Another study focusing on a *BRCA1* pathogenic missense variant found it to be ~500-fold enriched in Orkney compared to the UKB participants and doubled the number of kindreds in which the pathogenic variant was seen to segregate compared to what was previously known to the NHS^13^, highlighting again the advantages of cohort sequencing compared to familial cascade genetic testing.

Our work is a computational study of population genetic data that is not intended to provide insights into the aetiology or treatment of diseases. It aims to gauge the extent to which rare, deleterious variants are enriched in regional British and Irish populations. We demonstrate that, even with conservative thresholding, many regional populations are relatively enriched for otherwise very rare deleterious variants within genes that are already known to cause a range of rare human diseases. To our knowledge, these enrichments have not been observed previously and they provide useful insights into the regional genetic legacies of recent population dynamics. In addition, the deleterious variants we discover are potentially actionable, by inclusion in genetic screening efforts such as these that already exist for known isolated populations. Examples include screens tailored for individuals with AJ heritage in the UK^29^, the USA^45^, and Israel^46^, those from Old Order Amish and Old Order Mennonite communities in the USA^47^ and a recent small pilot trial offering testing for the *BRCA1* variant in the Orkney outer isle of Westray discussed above (https://www.nhsgrampian.org/news/2023/july/testing-pilot-trial-now-underway-for-orkney-cancer-gene-link/).

We believe that after careful consideration reproductive carrier screening could be carried out in a cost-effective manner, with a better understanding of the regional landscape of pathogenic rare variation across the British Isles, to which we have contributed. In the future, this landscape could inform new screening strategies, benefitting from the diverse regional burdens of pathogenic variation within a country, to decrease the burden of Mendelian disease.

## Discussion

The work described here is based on the UKB WES data for the first ~200,000 participants released in October, 2020^24^. We complemented these data with Viking Genes WES data for more than 4000 individuals from Shetland and Orkney (the Northern Isles) in Scotland, which were generated by the same provider (Regeneron Genetics Center) using the same Original Quality scores Functional Equivalence (OQFE) protocol as for the UKB dataset, to compile a combined dataset of 44,696 unrelated individuals born in various geographical regions (Methods). Based on the availability of sufficient numbers of UKB individuals with WES data, we divided individuals into 18 geographical regions: two representing the Northern Isles, three Scottish regions, two Welsh regions, ten English and one Irish region, consisting of individuals who self-identify as Irish and were born in either Northern Ireland or the Republic of Ireland (Methods). Additionally, based on the available genetic and genomic data, we identified the presence of individuals with Jewish heritage in the UKB dataset. We present several lines of evidence supporting our hypothesis these are mainly individuals of Ashkenazi Jewish (AJ) origin and which we define as full and part AJ individuals based on the degree of their Ashkenazi Jewish heritage, for a total of 20 genetic groups considered in our study.

Previous research showed a remarkable correlation between genomic variation and the geography of the UK and Ireland^5,6,26^. Here, we demonstrate that this signal is largely preserved in protein-coding exons in spite of the general intolerance to variation of these regions. Our results (Fig. 1) show a clear separation of the isolated Shetland and Orkney Isles populations as well as AJ individuals from each other and the other regions, and we recapture patterns consistent with reports of a Welsh-English-Scottish-Irish cline from previous studies based on genome-wide genotyping arrays^5,6^. Our analyses also rediscovered the distinction between individuals born in north and south Wales, and some level of distinctiveness exhibited by southeast Scotland individuals^5^. These findings should be considered when performing association studies, where accounting for population structure is of crucial importance. The geographical variation in genotypes in the UKB has been documented before in the context of GWAS studies^48,49^, our results suggest similar care must be taken in future exome-wide association studies based on UKB data.

Our analyses suggest that the observed regional structure is mainly driven by variants that are relatively rare (MAF < 5%) in our dataset of 10,001 unrelated individuals selected from the 20 regions, and that constitute ~90% of the detected variation. As a result of various factors shaping regional genetic landscapes, including effective population size, strength of purifying selection and gene flow, some of these variants drifted to different frequencies in different regions. We rediscover the strong influence of genetic drift in shaping variation in the Northern Isles of Scotland and among those with full or partial Ashkenazi Jewish (AJ) ancestry. For full AJ, almost half the known rare exonic variants (45%) are at least two-fold more or less frequent than in a Europe-wide reference sample, while the degree of variant frequency differences in Shetland and Orkney are comparable to part AJ (19%, 17%, 16%, respectively). The proportion of SNP loci exhibiting substantial frequency drift in each group correlates well with the assumed degree of geographical/cultural/social isolation, being highest in AJ and the Northern Isles, followed by Irish, Welsh, Scottish and English regions, but noticeable even in the cosmopolitan London region (Fig. 3, Supplementary Table 6).

The most important practical implication of the observed regional variation, resulting from various geographical/cultural/social barriers, is the elevated frequency of some variants with proven links to human health. In our study, we focused on significantly enriched exonic variants otherwise rarely or not found at all in a healthy control dataset (gnomAD MAF_NFE_ < 1%), predicted to be functional and previously reported as implicated in monogenic disorders (Methods). Applying stringent filtering criteria, we found 67 such unique variants which are at least five-fold enriched in one or more of the considered groups compared to NFE in gnomAD, with 95% of these variants being present, but extremely rare in gnomAD (90% of them with gnomAD MAF_NFE_ < 0.0004, i.e. 1/2500, Fig. 2) and the remaining 5% not found in any gnomAD population. Our analyses based on random mating suggest that the regionally enriched and potentially deleterious variants can be expected to result in a significant number - tens or hundreds - of homozygous individuals affected by a recessive genetic medical condition, which highlights the importance of future research into regional variation across the UK and Ireland, to inform effective genetic screening and counselling.

Our results provide a convincing illustration of the existence of some regionally enriched rare deleterious exonic variation in the UK and Ireland, whose effect may be otherwise overlooked if performing the analysis at country or nation level or even by only including participants from the large metropolitan areas. Due to the stringent filtering criteria adopted by us, the list of the 67 enriched variants is far from complete and due to the nature of the UKB data the variant frequency estimates are far from robust. However, we view our findings as a starting point which clearly warrants future research into regional variation in the UK and Ireland with the ultimate goal of designing a cost-efficient pan-UK genetic test including rigorously identified regionally enriched variants of medical importance.

There are several key issues that need to be addressed towards this goal. Firstly, our results are based on the analysis of 44,696 unrelated UKB individuals with WES data available, which constitutes <0.1% of the overall UK and Republic of Ireland population. In order to accurately estimate the regional frequency of these extremely rare deleterious exonic variants, a deeper, wider, more ethnically diverse and as random as possible sampling would be beneficial. This point is further illustrated by the observation that for five of the six regions in our study with no enriched variant discovered - East Anglia, Kent, England SW, Scotland NE and Hampshire&Wiltshire—the number of unrelated individuals included in our study for these regions is below the median number of participants for the remaining 14 regions. It can be expected that the release of the next UKB WES tranche of 500 k individuals would alleviate this problem to a certain extent, as well as increasing the breadth of regional coverage. The availability of such information will help in better understanding of the geography of the variant enrichments. For example, are the variants discovered to be enriched in South Wales region-specific, or are some of them also enriched in the neighbouring Welsh Marches (e.g. Gloucestershire, Herefordshire, Shropshire and Cheshire, in England), for which at the moment we lack sufficient UKB participants with WES data available? All these facts highlight an important limitation of our study as is, namely no firm conclusions can be drawn by comparing the numbers of enriched variants discovered across regions (as coverage is so variable and precise estimation of regional effective population sizes are not available yet). In addition, despite the inclusive efforts embedded in the UKB recruitment, there is participation bias towards older (median age at assessment = 58), healthier individuals from more economically affluent areas (median Townsend deprivation index score = −2.2) who self-identify as “White” (93.7%)^24,50^, and who live near the 22 UKB assessment centres, 17 of which were in England and none of which were in Northern Ireland, making the dataset not fully representative of the overall UK population.

Secondly, our strategy for identifying regionally enriched deleterious variants favours specificity over sensitivity. For example, our decision to consider only missense variants with CADD score ≥30 leads to omitting the rs76763715 variant, a common pathogenic variant reported in the homozygous and compound heterozygous state in individuals with Gaucher disease (type I). This condition exhibits a higher prevalence among individuals with Ashkenazi Jewish heritage and in our data the variant is 13 times more frequent in AJ individuals compared to London, but was excluded due to a CADD score of 24. Next, our chosen threshold of considering as enriched only regional variants observed with frequency at least 5 times higher compared to Non-Finnish Europeans is not necessarily optimal (e.g. the 3-M syndrome variant); a lower, or even no such threshold, may be more relevant from a medical perspective, highlighting the crucial importance of close collaboration between genetic scientists, clinicians, stakeholders and policy-makers. Further, in assessing the predicted effect of the discovered enriched variants, we retained only variants with substantial evidence of being pathogenic/likely pathogenic available as reported by ClinVar; however only about 20% of all enriched variants identified in our study were found with any pathogenicity annotation in ClinVar. Finally, in estimating the practical impact of the regionally enriched variants we focused on calculations of the genetic prevalence of individuals homozygous for these variants. This leads to a clear underestimation of individuals affected by the corresponding medical condition, due to the general genetic and locus heterogeneity of the monogenic recessive disorders and highlights the need for an additional, disorder-centric, systematic investigation of the regional frequency of all other known related variants.

## Methods

### Ethics statement

All participants in the Viking Health Study - Shetland (VIKING) gave written informed consent for broad ranging health and ancestry research including, whole genome/exome sequencing and the study was given a favourable opinion by the South East Scotland Research Ethics Committee (REC Ref 12/SS/0151). All participants in the Orkney Complex Disease Study (ORCADES) gave written informed consent for broad ranging health and population research, including sequencing and the study was approved by Research Ethics Committees in Orkney, Aberdeen (North of Scotland REC), and South East Scotland REC, NHS Lothian (reference: 12/SS/0151).

### Participant selection

The Viking Health Study—Shetland (VIKING)^9^ and Orkney Complex Disease Study (ORCADES)^25^ are family-based, cross-sectional studies that seek to identify genetic factors influencing cardiovascular and other disease risk in the population isolates of the Shetland and Orkney Isles in northern Scotland. These studies are now subsumed, along with VIKING II, under the Viking Genes umbrella (https://viking.ed.ac.uk/). 2105 participants were recruited to VIKING between 2013 and 2015, most having at least three grandparents from Shetland, while 2078 participants were recruited to ORCADES between 2005-2011, most having three or four grandparents from Orkney, the remainder with two Orcadian grandparents. Fasting blood samples were collected and many health-related phenotypes and environmental exposures were measured in each individual. The sequencing data generated for the Viking Genes project has been used already in several case studies showcasing the potential value of isolate population-based research resources for genomic medicine^9,13^.

#### Northern Isles

As part of Viking Genes (https://viking.ed.ac.uk/), >4000 individuals from the Shetland and Orkney islands (the Northern Isles of Scotland) were selected for whole exome sequencing. Since the focus of this work is on regional variation, we utilized the rich genealogical information collected as part of Viking Genes and from the 2134 Shetland participants with WES data we selected for further analysis the 1454 individuals with all four grandparents also born on the Shetland archipelago. To alleviate familial effects and to obtain a more representative snapshot of the regional variation, we identified related individuals up to first cousins once removed and closer and equivalents (PLINK v1.90b4^51^; pi_hat ≥ 0.0625) and generated the maximum unrelated set (using PRIMUS v1.9.0^52^) of 492 unrelated Shetlanders with all four grandparents born on the Shetland archipelago. Similarly, from the 2092 Orkney participants with WES data we selected a maximum unrelated set of 509 unrelated Orcadians with all four grandparents born on the Orkney archipelago.

#### UKB

The UK Biobank Exome Sequencing Consortium (UKB-ESC) is a private-public partnership between the UKB and eight biopharmaceutical companies that will complete the sequencing of exomes for all ~500,000 UKB participants^24^. To explore the regional variation, we considered geographical region of birth based on historic counties and selected only those regions for which there were at least 500 UKB participants with WES data available, using the 200 k WES UKB tranche (released in October 2020) after excluding all participants who withdrew their consent. Since grandparents’ birthplace information is not available for the UKB participants, in order to focus on the regional variation in the UKB data we selected only participants who self-identify as “White British” (UKB field: 21000), exhibit very similar genetic ancestry based on a principal components analysis of the UKB whole-genome SNP array genotypes (UKB field: 22006) and who were born outside large metropolitan areas in the corresponding region. The participants satisfying the above criteria for each region were then evaluated for relatedness and the maximum unrelated set per region generated as for the Northern Isles cohorts.

The three Scotland regions we included in our study are: Scotland North East (Aberdeen, Aberdeenshire, Kincardineshire, Angus, Banffshire, Dundee, Fife, Perthshire, Kinross-shire, Clackmannanshire, Stirlingshire, Moray) with 1680 unrelated individuals, Scotland South East (East Lothian, Midlothian, West Lothian, Selkirkshire, Berwickshire, Roxburghshire, Peeblesshire; excluding Edinburgh) with 667 unrelated individuals and the south-western region of Strathclyde (Lanarkshire, Renfrewshire, Dunbartonshire, Ayrshire; excluding Glasgow) with 2077 unrelated individuals (Supplementary Table 1).

The two Wales regions we included in our study are: Wales North (Anglesey, Caernarfonshire, Merionethshire, Montgomeryshire, Flintshire, Denbighshire) with 883 unrelated individuals and Wales South (the remaining part of Wales; excluding individuals born in a 5 mile radius area centred on Cardiff) with 3239 unrelated individuals.

The ten English regions we included in our study are: England North East (Northumberland and Durham; excluding individuals born in a 15 mile radius area centred on Newcastle-upon-Tyne) with 2982 unrelated individuals, Yorkshire (excluding individuals born in a 5 mile radius areas centred on Kingston-upon-Hull and Doncaster and 15 mile radius areas centred on Leeds, Bradford and Sheffield) with 3276 unrelated individuals, Lancashire (excluding individuals born in two 15 mile radius areas centred on each of Liverpool and Manchester) with 3007 unrelated individuals, Nottinghamshire with 4192 unrelated individuals, Staffordshire (excluding individuals born in a 10 mile radius areas centred on Birmingham and Wolverhampton) with 3526 unrelated individuals, East Anglia (Norfolk and Suffolk) with 923 unrelated individuals, Hampshire and Wiltshire (excluding individuals born in a 5 mile radius areas centred on Portsmouth and Southampton) with 1925 unrelated individuals, Kent (excluding individuals born in a 17 mile radius area centred on the City of London) with 1327 unrelated individuals and England South West (Cornwall and Devon) with 1412 unrelated individuals. We also included Central London (individuals born in a 10 mile radius area centred on the City of London) with 8913 unrelated individuals, which given the cosmopolitan nature of the capital are expected to serve as a useful control in terms of regional variation.

We have also included in our analysis as Irish a group of 2005 unrelated individuals who self-identify as Irish (UKB field: 21000) and born in either Northern Ireland or the Republic of Ireland (UKB field: 1647).

The last group of UKB participants included in our study are individuals of Ashkenazi Jewish (AJ) ancestry, split into full AJ (1004 unrelated individuals) and part AJ (657 unrelated Individuals).

### Sequencing, mapping and variant calling

The WES sequencing, read mapping and variant calling for the ~200 k UKBB participants was performed following the OQFE protocol^24^. The WES sequencing, read mapping and variant calling for the individuals from the Northern Isles (Shetland and Orkney) was performed at the same sequencing facility using the same sequencing and data processing protocols as for the UKBB participants. The starting point for our analyses were the project VCF files generated by the OQFE protocol.

### Variant QC and annotation

Using the unrelated individuals selected for each of the 20 regions described above, we generated 20 regional VCFs by extracting from the corresponding project data only autosomal variation present in the particular region, excluding non-variant sites and sites with >10% missing genotypes. The regional VCF was decomposed and normalized and the remaining missing genotypes (‘./.’) were set to homozygous reference genotype (‘0/0’). All variants in the 100 bp flanking regions outside the capture region were excluded, as well as variants in low-complexity regions based on sdust^53^ or failing the filtering criteria in gnomADg v3.1.1^30^.

Further, any individual SNPs with read depth (DP) < 7, genotype quality (GQ) < 10, heterozygous SNPs with variant allele frequency (VAF) < 0.15 or VAF > 0.85, homozygous SNPs with VAF < 0.85 and any SNPs in windows with problematic gnomADg v3.1.1 coverage (defined as 10 bp windows centred on any base with coverage < 10x) were excluded. Similarly, any individual INDELs with DP < 10, GQ < 10, heterozygous INDELs with VAF < 0.2 or VAF > 0.8 and homozygous INDELs with VAF < 0.8, are excluded. The information about the number of variants filtered at each step is reported in Supplementary Table 9.

The variants in the resulting VCF were annotated with their predicted functional effect using VEP^54^ (v102) including annotation of each variant with its MAF as reported in gnomADg v3.1.1. Lastly, we excluded any variant if it has not been observed in gnomADg v3.1.1 but is detected in the regional VCF with MAF ≥ 10%.

### MDS and UMAP analysis

Our MDS and UMAP analyses of the 10,001 unrelated individuals (492 Shetlandic, 509 Orcadian and 500 randomly chosen individuals from the remaining 18 regions) are based on their autosomal coding SNP variation. We considered only SNPs also found in gnomAD in order to alleviate the potential effect of false positive variation in our data. From the assembled VCF, we excluded all variants in regions with known long-range high linkage disequilibrium^55,56^, as well as all singleton variants. From the remaining 512,327 SNPs we selected only biallelic variants with observed AF in our dataset of 10,001 unrelated individuals of <5%. The selected 465,647 SNPs (91.8% of all) were screened and pruned based on further linkage disequilibrium evidence (r^2^ < 0.02; PLINK 1.90b4 with --indep-pairwise 500 5 0.2) resulting in a final set of 401,895 markers which we used as an input for our Multi-Dimensional Scaling (MDS) analysis performed with PLINK (--cluster --mds-plot 50, Supplementary Fig. 3A). The UMAP analysis (Fig. 1A) was performed on the top 20 MDS dimensions using the general_umap_script.py^18,57^. The same approach and parameter values were used for analysing the data from the 8000 mainland UK individuals (Fig. 1B).

### Unrooted NJ phylogenetic tree of the 20 regions based on *F*_*ST*_ distances

A commonly used metric for evaluating the similarity between populations is the Weir and Cockerham *F*_*ST*_ fixation index^58^, which is a measure of population differentiation due to genetic structure and represents the relative amount of genetic variance between populations compared to the total genetic variance within these populations^59^. We calculated the pair-wise *F*_*ST*_ distances among the 20 regions based on non-singleton known SNPs with MAF < 5% in our chosen set of 10,001 unrelated individuals using the --weir-fst-pop option in VCFtools^60^ (v0.1.13) with window size of 1 Mb and window step of 250 kb (Supplementary Table 2). For each of the full AJ, part AJ, Shetland and Orkney regions, we computed the mean *F*_*ST*_ distance to the 16 mainland regions as the average of the region’s distances to the mainland ones; for a mainland region itself, we computed its mean *F*_*ST*_ distance to the mainland as the average of the region’s distances to the remaining 15 mainland regions (Supplementary Fig. 5). A similar approach was used for computing the mean *F*_*ST*_ distances within and across nations. For constructing the phylogenetic tree we used PHYLIP^61^ (v3.697) in Neighbour-Joining (NJ) mode using full AJ as outgroup and randomized the region input order. The computed tree is visualized with iTOL^62^.

### Regionally enriched deleterious variation

In order to increase our discovery power, we based our search for regionally enriched potentially deleterious variants on the full subsets of unrelated individuals per each region (Supplementary Table 1). To focus on functional variation, our analysis is restricted to variants on canonical transcripts with VEP predicted stop gained, start lost, splice donor/acceptor site (the last 2 bp at each end of the intron), stop lost and frameshift effects, as well as missense and splice region variants (1–3 bp into an exon or 3–8 bp into an intron) for which we imposed the additional criterion of having CADD score ≥ 30. From these variants, we selected as enriched variants those that exhibit regional frequency at least 5 times higher than the variant’s frequency observed in the genomes of 34,029 Non-Finnish Europeans (NFE) in gnomAD^30^ (v3.1.1) and the enrichment being statistically significant (Fisher’s Exact Test, one-sided, Bonferroni adjusted for multiple testing). Variants with regional MAF ≥ 5% (MAF_NFE_ ≥ 1%) are excluded as less likely to be implicated in monogenic disorders, as well as variants seen in <5 unrelated individuals within region, in order to focus on regional, rather than familial variation. Lastly, we screened the regionally enriched variants against ClinVar^31^ and selected only those reported in ClinVar to be pathogenic/likely pathogenic with criteria provided, multiple submitters and no conflicting interpretation (at least 2 star variants) and further manually curated by us to select only variants explicitly reported in peer-reviewed publication(s).

### Nucleotide diversity analysis

The nucleotide diversity analysis was performed at variant site level, using VCFtools^60^ (v0.1.13) using the --site-pi option and using as markers the 44,108 SNP variants with MAF > 5% in the dataset of 10,001 unrelated individuals from 20 regions which are also present in the full gnomAD dataset.

### Reporting summary

Further information on research design is available in the Nature Portfolio Reporting Summary linked to this article.

## Supplementary information

Supplementary Information

Reporting Summary

## Data Availability

For ORCADES and VIKING, the research data used in this study and/or DNA samples are available through managed access by application (accessQTL@ed.ac.uk), following approval by the QTL Data Access Committee with expected timeframe for response of about 2 months (https://viking.ed.ac.uk/). These data are available under managed access due to the consent given by the participants and Research Ethics Committee approvals. Each approved project is subject to a data or materials transfer agreement (D/MTA) or commercial contract. Data may be shared with academic or commercial recipients worldwide and may be used within the parameters of the Research Ethics Committee approvals. The UK Biobank genotypic data used in this study were approved under application 19655 and are available to qualified researchers via the UK Biobank managed data access process. All other data supporting the findings described in this manuscript are either from publicly available resources (e.g., gnomAD, ClinVar, CADD scores) or included in the article and its Supplementary Information files.
