# Supplementary material for: Regionally enriched rare deleterious exonic variants in the UK and Ireland
Source: Nat Commun. 2024 Oct 2;15:8454. doi: 10.1038/s41467-024-51604-2 (PMC11446911; doi:10.1038/s41467-024-51604-2)
Supplement: Supplementary file 1 — Supplementary Information [file 41467_2024_51604_MOESM1_ESM.pdf]

Regionally enriched rare deleterious exonic variants in the UK and Ireland

Mihail Halachev[1,+], Viktoria-Eleni Gountouna[1], Alison Meynert[1], Gannie Tzoneva[2], Alan R. Shuldiner[2], Colin A. Semple[1,^], James F. Wilson[1,3,4,^]

[1] MRC Human Genetics Unit, Institute of Genetics and Cancer, University of Edinburgh, United Kingdom

[2] Regeneron Genetics Center, Tarrytown, NY, USA

[3] Centre for Global Health Research, Usher Institute, University of Edinburgh, United Kingdom

[4] Centre for Genomic and Experimental Medicine, Institute of Genetics and Cancer, University of Edinburgh, United Kingdom

[+] Corresponding author (mhalache@exseed.ed.ac.uk)

[^] These authors jointly supervised this work

Supplementary Figures

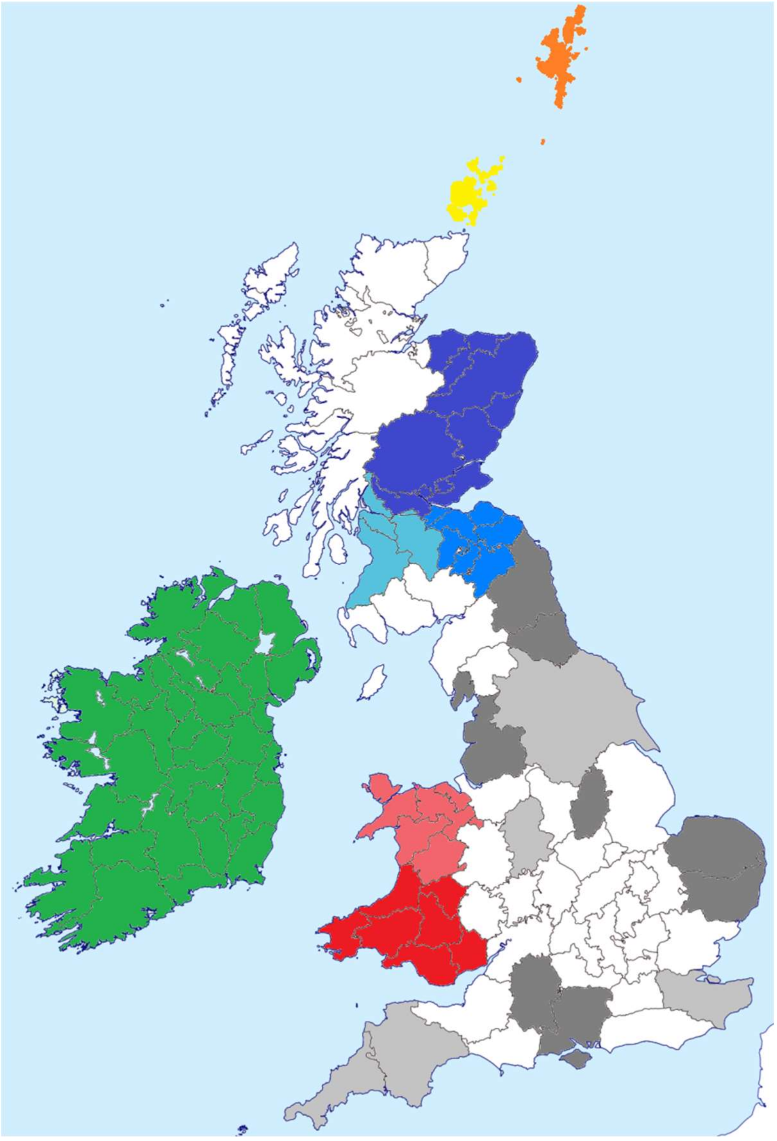

Figure S1. Regions of origin of the participants in this study

The participants from the Northern Isles of Scotland (Shetland in orange and Orkney in yellow) are obtained from the Viking Genes project (https://www.ed.ac.uk/viking). The three mainland UKB Scotland regions (in blue) we included in our study are Scotland North East, Scotland South East and the south-western region of Strathclyde. The two Wales regions (in red) we included in our study are Wales North and Wales South. The ten English regions (in grey) we included in our study are England North East, Yorkshire, Lancashire, Nottinghamshire, Staffordshire, East Anglia, Hampshire and Wiltshire, Kent and England South West. We also included Central London (individuals born in a 10 mile radius area centred on the City of London). Irish participants were selected based in self-identification as Irish and born in either Northern Ireland or the Republic of Ireland (in green). The last group of UKB participants included in our study are individuals of Ashkenazi Jewish (AJ) ancestry, split into full and part AJ, regardless of their geographical region of origin. The county boundary file (ESRI Shapefile) was downloaded from the OS Data Hub for public sector (https://osdatahub.os.uk/downloads/open/BoundaryLine). Contains OS data © Crown copyright and database rights [2024], licensed under the Open Government Licence v3.0.

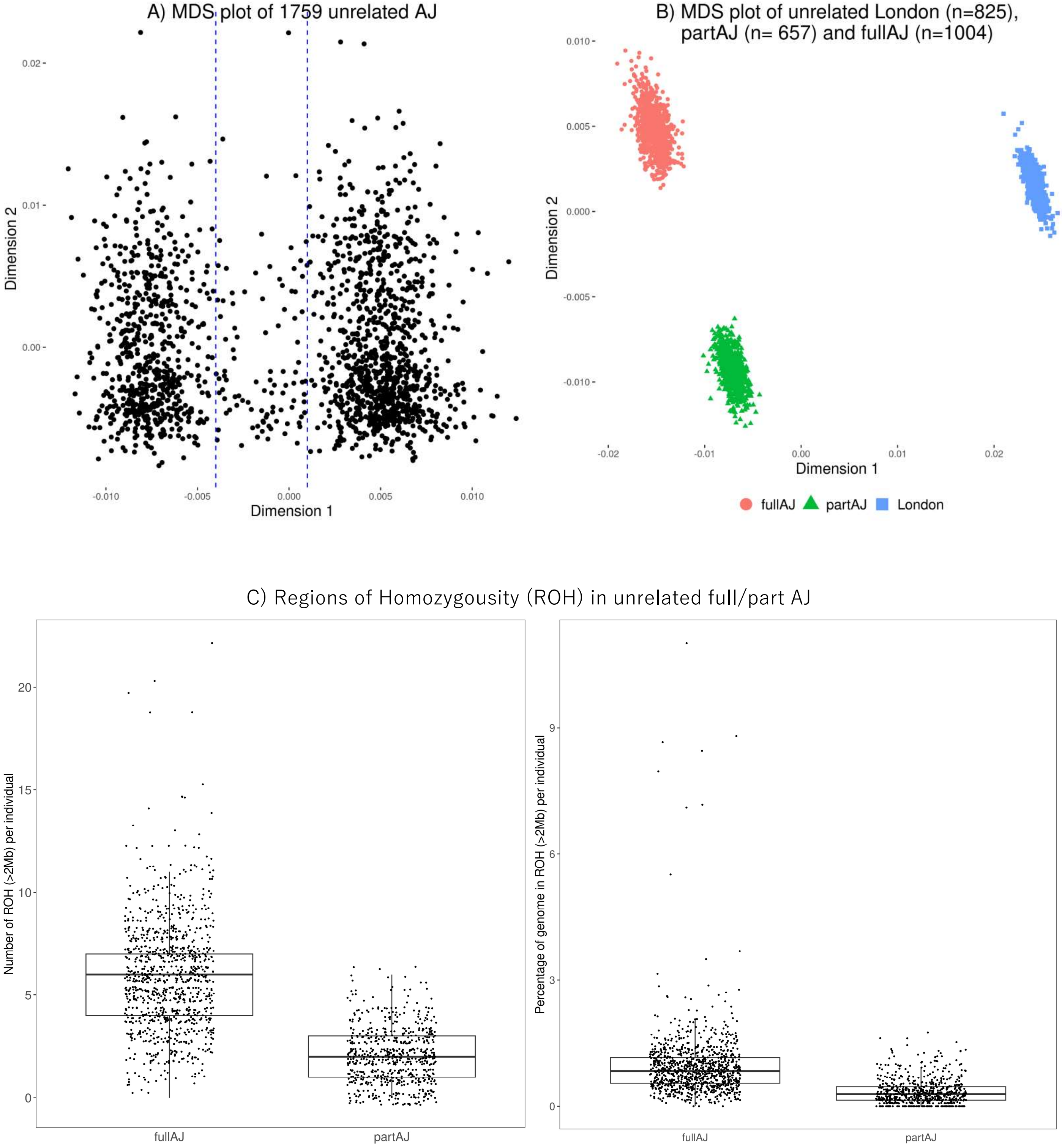

Figure S2. Analyses of unrelated Ashkenazi Jewish individuals' heritage

A) MDS plot based on biallelic, non-singleton and linkage-disequilibrium (LD) pruned known SNPs (MAF > 1%) in 1759 unrelated AJ individuals reveals two main clusters speculated to represent different level of admixture between AJ and general UK population. The main reason why instead of continuous spectrum, we observe roughly two main clusters, with only a small number of individuals placed in between is that a continuous spectrum can only be expected when there has been thorough admixture for at least several generations. For example, it can be estimated that in order for an admixed individual with 3/8^{ths} or 5/8^{ths} AJ heritage (requires at least two generations, ~60 years) to be present in the UKB data (median age of participants is 58 years), the admixture process should have started more than 100 years ago, i.e. only a single generation after the main migration of Russian and Eastern European Jewish to the UK between 1870 and 1914; **B)** MDS plot of the selected individuals from the two main AJ clusters (left and right from the vertical dashed blue lines in panel A) coupled with 825 unrelated Londoners based on biallelic, non-singleton and LD pruned known SNPs (MAF > 1%) ; **C)** Analyses confirm full AJ individuals exhibit higher number/larger proportion of genome in ROH compared to part AJ. For the boxplots, center line: median; box limits: upper and lower quartiles; whiskers: 1.5x interquartile range.

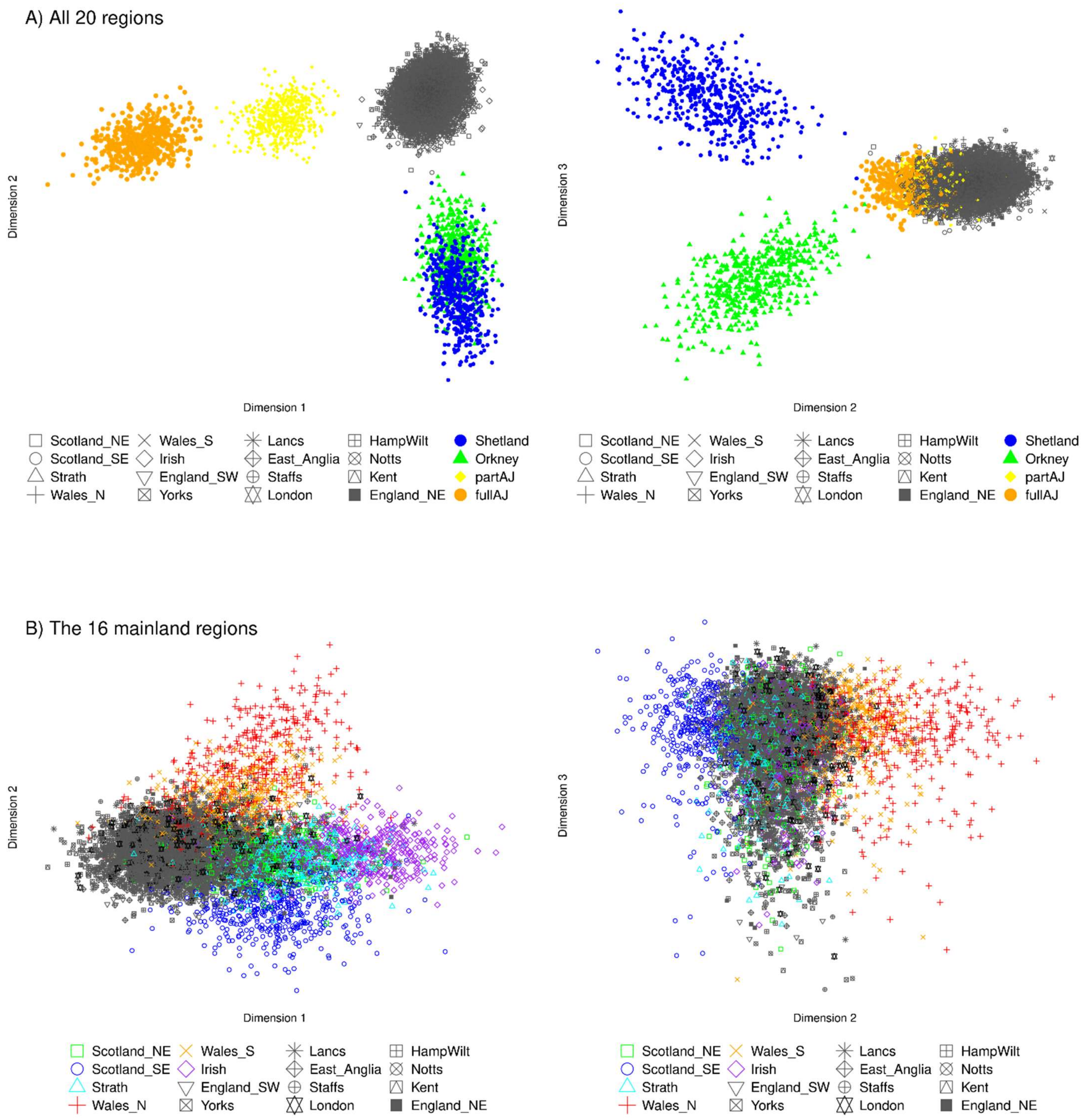

Figure S3. Distinction among regional populations based upon MDS analysis of rare exonic variation.

The regional MDS analyses are based on biallelic, non-singleton and LD pruned known SNPs with MAF < 5% in the considered groups of unrelated individuals (n = 10,001 for all 20 regions and n = 8,000 for the 16 mainland regions); the top 20 of the discovered MDS dimensions are subsequently used as input to the corresponding UMAP projections (Fig 1).

A) MDS plot for all 20 groups in our study illustrating the distinction between AJ and the remaining 18 groups with part AJ group placed between full AJ and general UK population (dimension 1), the genetic distinctiveness of the Northern Isles (dimension 2) and the genetic dissimilarity between Shetlandic and Orcadian individuals (dimension 3); **B)** MDS plot for the 16 mainland regions, with dimension 1 capturing the Irish-to-Scottish/Irish-to-English difference and dimension 2 separating North Wales from South East Scotland.

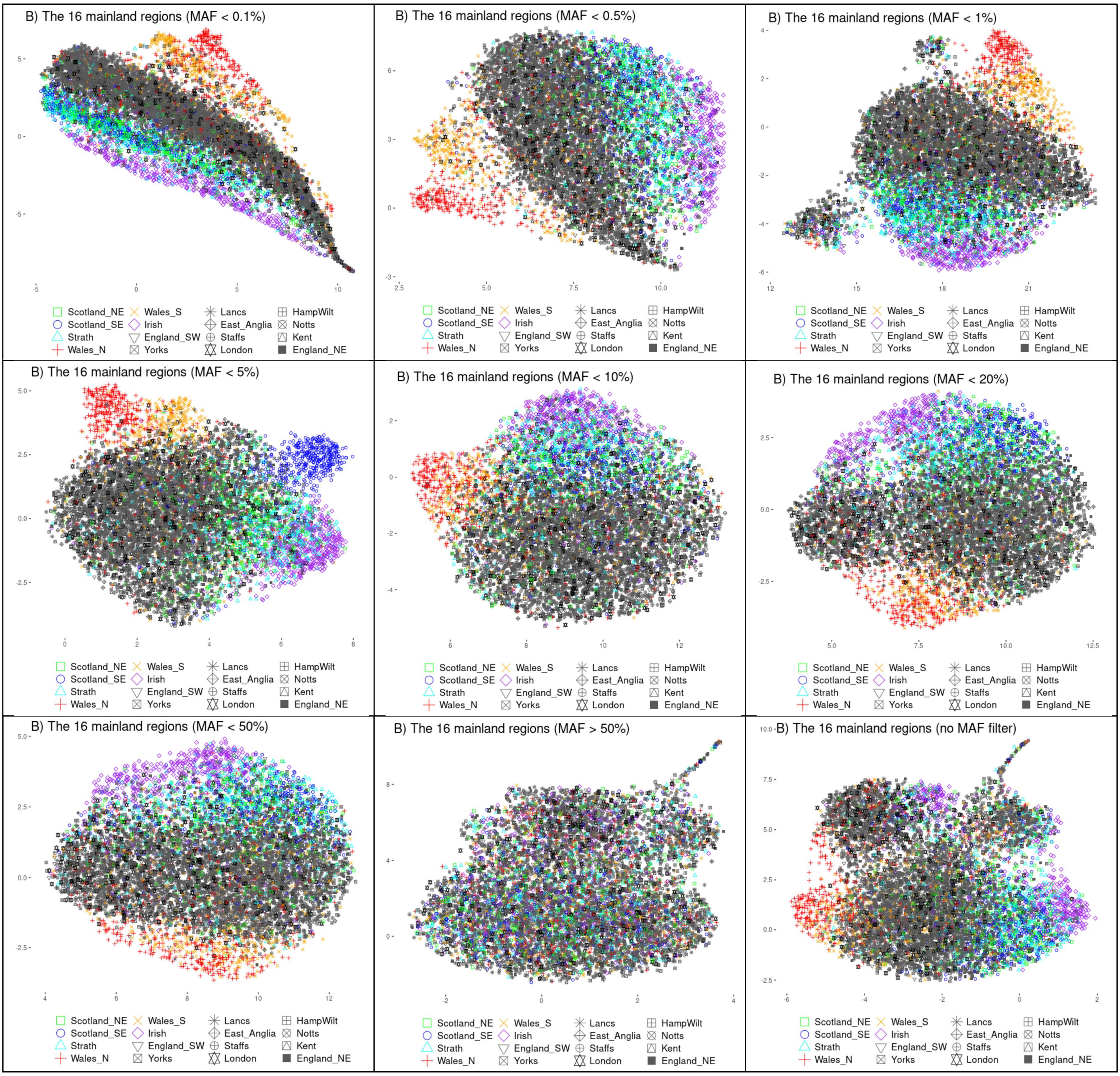

Figure S4. The effect of the selected MAF threshold in computing the UMAP projections of the 16 mainland regions (Fig 1B).

While any MAF threshold < 50% allows for re-discovering the Irish-Scottish-English-Welsh cline previously reported based in while genome genotyping array data, iterative analyses of our whole-exome sequencing data suggest the MAF < 5% threshold provides the best UMAP resolution by additionally recapitulating the known South Wales, North Wales and South East Scotland distinctiveness.

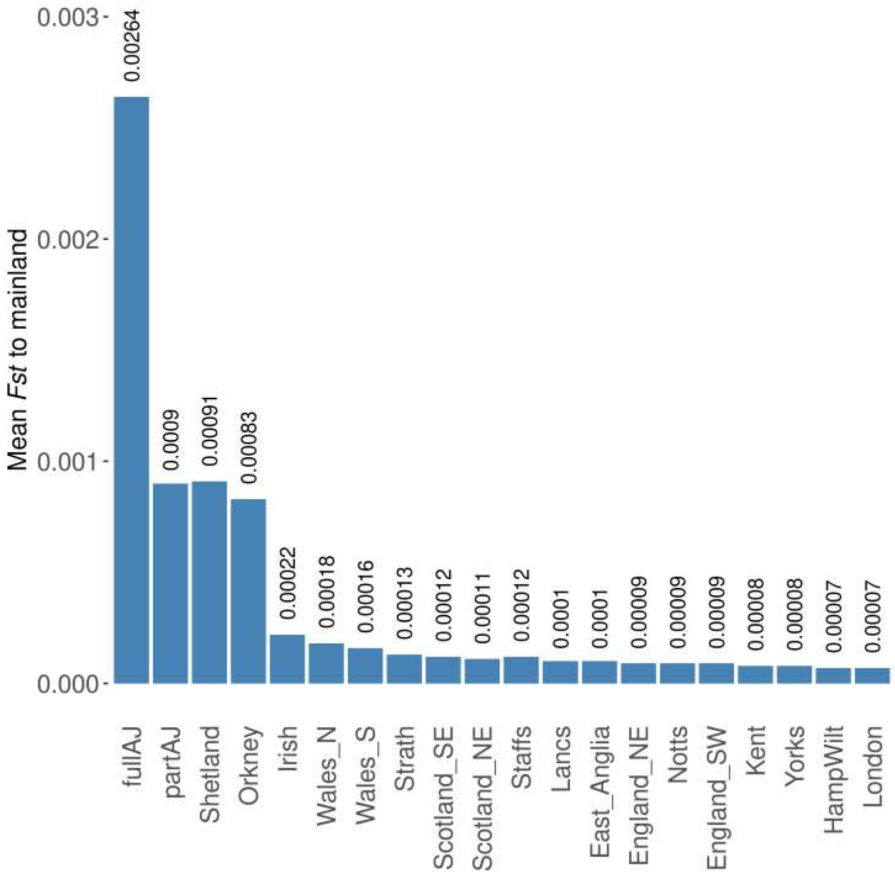

Figure S5. The mean F_{ST} distance for each region to the 16 mainland regions (excluding the Northern Isles and AJ). All pair-wise F_{ST} distances between the 20 regions are presented in Table S2 (below).

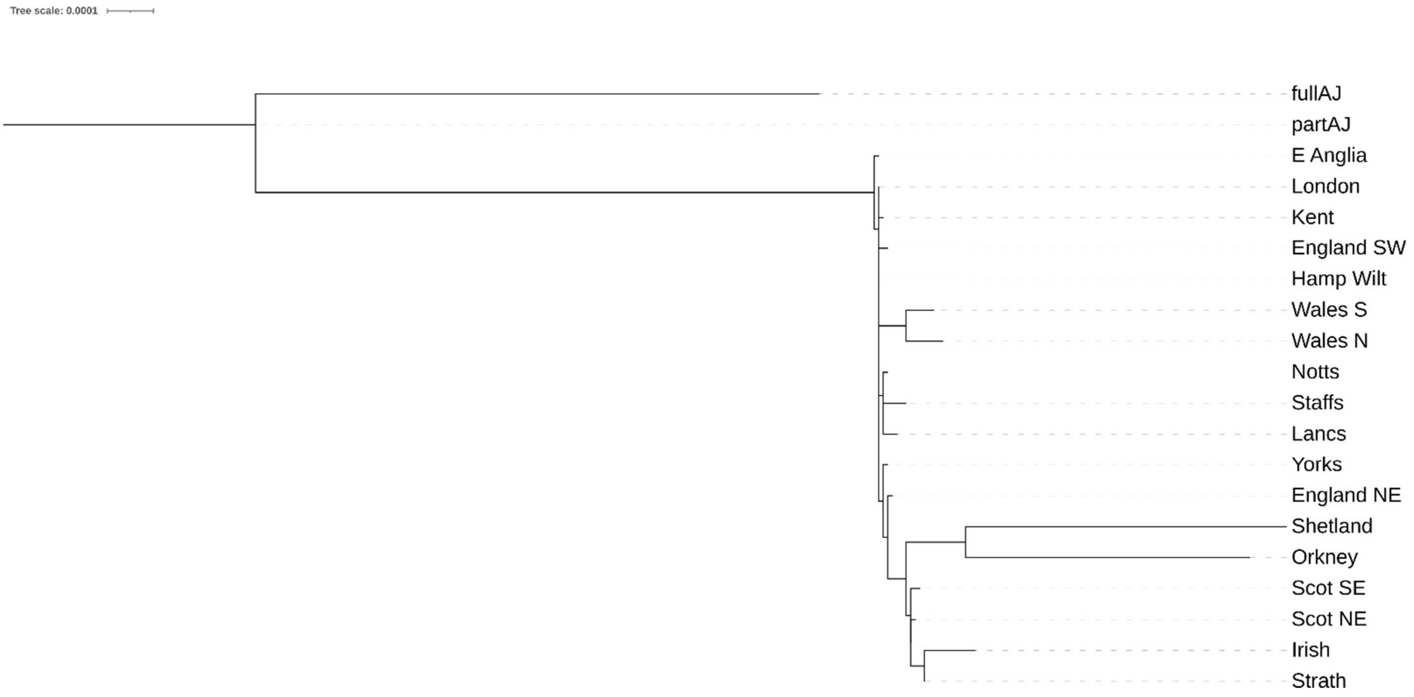

Figure S6. NJ phylogenetic tree for the 20 regions built based upon the pair-wise F_{ST} distances

Supplementary Tables

Table S1. Median (IQR) counts of ALT (non-reference) alleles per individual

		SNP						INDEL					
		**ultra-rare**		**known**			**total**	**ultra-rare**		**known**			**total**
**region**	**n**	**singleton**	**shared**	**very rare**	**rare**	**common**		**singleton**	**shared**	**very rare**	**rare**	**common**	
full AJ	1004	11 (0)	2 (0)	1,037 (5)	1,172 (5)	30,016 (23)	32,238	0 (0)	0 (0)	31 (1)	34 (0)	765 (2)	830
part AJ	657	27 (1)	1 (0)	841 (4)	1,146 (4)	30,057 (23)	32,072	1 (0)	0 (0)	27 (1)	33 (1)	768 (2)	829
Shetland	492	16 (0)	32 (0)	618 (4)	1,127 (4)	30,071 (18)	31,864	1 (0)	2 (0)	20 (1)	34 (1)	764 (2)	821
Orkney	509	17 (1)	22 (1)	621 (3)	1,120 (4)	30,102 (19)	31,882	1 (0)	1 (0)	21 (1)	33 (1)	764 (2)	820
Scotland NE	1680	33 (1)	6 (0)	627 (3)	1,119 (4)	30,099 (21)	31,884	1 (0)	0 (0)	20 (0)	32 (0)	770 (2)	823
Scotland SE	667	34 (1)	3 (1)	627 (4)	1,122 (4)	30,115 (17)	31,901	1 (0)	0 (0)	21 (1)	32 (1)	772 (1)	826
Strathclyde	2077	29 (0)	4 (0)	628 (3)	1,122 (4)	30,099 (21)	31,882	1 (0)	0 (0)	20 (0)	32 (0)	769 (2)	822
Wales N	883	34 (0)	4 (0)	630 (3)	1,125 (4)	30,103 (12)	31,896	1 (0)	0 (0)	20 (0)	32 (1)	770 (3)	823
Wales S	3239	32 (1)	9 (0)	628 (2)	1,119 (5)	30,109 (19)	31,897	1 (0)	1 (0)	20 (0)	32 (0)	773 (3)	827
Irish	2005	29 (1)	4 (0)	618 (3)	1,122 (5)	30,081 (19)	31,854	1 (0)	0 (0)	20 (0)	32 (0)	766 (2)	819
East Anglia	923	44 (1)	3 (0)	634 (3)	1,124 (4)	30,071 (17)	31,876	2 (0)	0 (0)	20 (0)	33 (0)	768 (2)	823
England NE	2982	34 (0)	8 (0)	633 (3)	1,124 (5)	30,106 (21)	31,905	1 (1)	0 (0)	21 (1)	32 (1)	772 (2)	826
England SW	1412	41 (1)	4 (0)	632 (3)	1,127 (5)	30,091 (22)	31,895	2 (0)	0 (0)	20 (0)	32 (1)	769 (2)	823
Hamp&Wilt	1925	42 (0)	3 (0)	631 (2)	1,124 (4)	30,087 (20)	31,887	2 (0)	0 (0)	20 (0)	33 (0)	769 (2)	824
Kent	1327	43 (0)	3 (0)	633 (3)	1,124 (5)	30,082 (17)	31,885	2 (0)	0 (0)	20 (0)	33 (1)	768 (2)	823
Lancs	3007	31 (1)	7 (1)	634 (3)	1,123 (4)	30,065 (16)	31,860	1 (0)	1 (1)	20 (1)	32 (1)	765 (3)	819
Notts	4192	34 (0)	11 (0)	635 (3)	1,121 (4)	30,077 (22)	31,878	1 (1)	1 (0)	20 (1)	32 (1)	768 (2)	822
Staffs	3526	27 (1)	15 (0)	636 (2)	1,124 (4)	30,102 (22)	31,904	1 (0)	1 (0)	20 (0)	32 (1)	771 (1)	825
Yorks	3276	36 (1)	7 (0)	633 (3)	1,122 (5)	30,100 (16)	31,898	2 (0)	0 (0)	20 (1)	32 (1)	770 (2)	824
London	8913	36 (1)	10 (0)	633 (2)	1,123 (4)	30,067 (21)	31,869	2 (1)	1 (0)	20 (0)	32 (0)	764 (2)	819

n: number of unrelated individuals per region; **ultra-rare** SNP/INDEL: variants not found in the gnomAD dataset (v3.1.1, containing data for 76156 genomes from unrelated individuals world-wide), singleton: variant found only in a single individual from this region, shared: variant found in more than one individual from this region; **very rare** SNP/INDEL: found in gnomADg v3.1.1 dataset with Non-Finnish European (NFE, n = 34,029 individuals, the closest available reference population for the White British individuals in our study) MAF < 1%; **rare** SNP/INDEL: 1% ≤ NFE MAF < 5%; **common** SNP/INDEL: NFE MAF ≥ 5%; **median and IQR** values for each region: based on random selection of 450 individuals (with replacement) repeated 10k times. The split to very rare, rare and common known variants was done for compatibility with existing population genetics literature; note that direct comparison between the 20 regions in our work based on the number of variants in these categories may not be fully informative due to reasons including the lack of suitable, publicly available reference dataset for individuals of AJ heritage (with partial West-Asian ancestry) and the potential regional imbalance of UK individuals whose data is included in the composition of the gnomAD NFE reference dataset. The computation of the number of ALT alleles per individual was performed as follows. Starting from the project VCF files generated by the UKB's OQFE protocol, we extracted the genetic data for the individuals in each of the 20 regions using *bcftools* and recomputing their AC, AN and AF tags. The 22 autosomal chromosomal VCFs per individual are then combined and filtered as described in the "Variant QC and annotation" section. The ALT allele counts per individual, their medians and IQR (Table S1) are computed with an in-house python script using the GT information, incrementing a counter by 1 for heterozygous and by 2 for homozygous variants encountered in this individual.

Table S2. Pair-wise F_{ST} estimates for the 20 UKB regions

	full AJ	part AJ	Shetland	Orkney	Irish	Wales N	Wales S	Strathclyde	Scotland SE	Scotland NE	Staffs	Lancs	East Anglia	England NE	Notts	England SW	Kent	Yorks	Hamp&Wilt	London
full AJ	n/a	0.00068	0.00382	0.00371	0.00288	0.00277	0.00275	0.00267	0.00266	0.00265	0.00263	0.00262	0.00256	0.00258	0.00257	0.00260	0.00258	0.00257	0.00257	0.00256
part AJ	0.00068	n/a	0.00172	0.00165	0.00107	0.00100	0.00097	0.00094	0.00093	0.00092	0.00090	0.00089	0.00084	0.00087	0.00085	0.00086	0.00085	0.00085	0.00084	0.00083
Shetland	0.00382	0.00172	n/a	0.00132	0.00101	0.00102	0.00101	0.00087	0.00087	0.00084	0.00094	0.00091	0.00090	0.00088	0.00090	0.00089	0.00089	0.00086	0.00087	0.00087
Orkney	0.00371	0.00165	0.00132	n/a	0.00091	0.00094	0.00092	0.00078	0.00078	0.00075	0.00087	0.00083	0.00082	0.00080	0.00082	0.00082	0.00081	0.00079	0.00079	0.00079
Irish	0.00288	0.00107	0.00101	0.00091	n/a	0.00029	0.00028	0.00012	0.00017	0.00015	0.00029	0.00024	0.00026	0.00020	0.00026	0.00023	0.00024	0.00021	0.00022	0.00020
Wales N	0.00277	0.00100	0.00102	0.00094	0.00029	n/a	0.00015	0.00022	0.00022	0.00020	0.00017	0.00017	0.00018	0.00017	0.00017	0.00017	0.00017	0.00016	0.00015	0.00014
Wales S	0.00275	0.00097	0.00101	0.00092	0.00028	0.00015	n/a	0.00020	0.00021	0.00019	0.00018	0.00017	0.00015	0.00015	0.00015	0.00013	0.00013	0.00014	0.00012	0.00012
Strathclyde	0.00267	0.00094	0.00087	0.00078	0.00012	0.00022	0.00020	n/a	0.00004	0.00004	0.00017	0.00014	0.00014	0.00009	0.00013	0.00013	0.00013	0.00010	0.00011	0.00011
Scotland SE	0.00266	0.00093	0.00087	0.00078	0.00017	0.00022	0.00021	0.00004	n/a	0.00004	0.00016	0.00014	0.00013	0.00008	0.00012	0.00011	0.00011	0.00009	0.00010	0.00010
Scotland NE	0.00265	0.00092	0.00084	0.00075	0.00015	0.00020	0.00019	0.00004	0.00004	n/a	0.00016	0.00012	0.00012	0.00008	0.00011	0.00010	0.00010	0.00008	0.00009	0.00008
Staffs	0.00263	0.00090	0.00094	0.00087	0.00029	0.00017	0.00018	0.00017	0.00016	0.00016	n/a	0.00008	0.00009	0.00010	0.00006	0.00009	0.00008	0.00008	0.00007	0.00007
Lancs	0.00262	0.00089	0.00091	0.00083	0.00024	0.00017	0.00017	0.00014	0.00014	0.00012	0.00008	n/a	0.00007	0.00007	0.00005	0.00008	0.00006	0.00005	0.00006	0.00006
East Anglia	0.00256	0.00084	0.00090	0.00082	0.00026	0.00018	0.00015	0.00014	0.00013	0.00012	0.00009	0.00007	n/a	0.00007	0.00004	0.00005	0.00004	0.00004	0.00003	0.00002
England NE	0.00258	0.00087	0.00088	0.00080	0.00020	0.00017	0.00015	0.00009	0.00008	0.00008	0.00010	0.00007	0.00007	n/a	0.00005	0.00006	0.00005	0.00003	0.00005	0.00004
Notts	0.00257	0.00085	0.00090	0.00082	0.00026	0.00017	0.00015	0.00013	0.00012	0.00011	0.00006	0.00005	0.00004	0.00005	n/a	0.00005	0.00003	0.00003	0.00003	0.00002
England SW	0.00260	0.00086	0.00089	0.00082	0.00023	0.00017	0.00013	0.00013	0.00011	0.00010	0.00009	0.00008	0.00005	0.00006	0.00005	n/a	0.00004	0.00005	0.00002	0.00002
Kent	0.00258	0.00085	0.00089	0.00081	0.00024	0.00017	0.00013	0.00013	0.00011	0.00010	0.00008	0.00006	0.00004	0.00005	0.00003	0.00004	n/a	0.00004	0.00002	0.00002
Yorks	0.00257	0.00085	0.00086	0.00079	0.00021	0.00016	0.00014	0.00010	0.00009	0.00008	0.00008	0.00005	0.00004	0.00003	0.00003	0.00005	0.00004	n/a	0.00003	0.00003
Hamp&Wilt	0.00257	0.00084	0.00087	0.00079	0.00022	0.00015	0.00012	0.00011	0.00010	0.00009	0.00007	0.00006	0.00003	0.00005	0.00003	0.00002	0.00002	0.00003	n/a	0.00001
London	0.00256	0.00083	0.00087	0.00079	0.00020	0.00014	0.00012	0.00011	0.00010	0.00008	0.00007	0.00006	0.00002	0.00004	0.00002	0.00002	0.00002	0.00003	0.00001	n/a

F_{ST} values
< 0.0001
0.0001-0.0002
0.0002-0.0003
0.0003 - 0.001
> 0.001

Table S3. Enriched and potentially deleterious variants in Ashkenazi Jewish samples.

MAF_{REG}: regional MAF of the variant

MAF_{NFE}: MAF of the variant in Non-Finnish European individuals in gnomAD

Conditions found primarily in Ashkenazi Jewish (*), in Sephardi-Mizrahi Jewish (^) and in all Jewish groups (+)

ClinVar Variant Allele ID	Gene	Condition	Region	MAF_{REG}	Enrichment (vs MAF_{NFE})
98339	*CPT2*	Carnitine palmitoyltransferase II deficiency*	full AJ	0.0070	474x
20541	*ADAMTS2*	Ehlers-Danlos syndrome dermatosparaxis type*	full AJ	0.0055	373x
29282	*MTTP*	Abetalipoproteinaemia*	full AJ	0.0045	305x
361210	*TECPR2*	Spastic paraplegia 49, autosomal recessive^	full AJ	0.0035	237x
19617	*BBS2*	Retinitis pigmentosa	full AJ	0.0035	237x
340082	*BLM*	Bloom syndrome*	full AJ	0.0030	203x
230781	*DNAI2*	Ciliary dyskinesia, primary, 9*	full AJ	0.0030	203x
447466	*ATP13A2*	Kufor-Rakeb syndrome	full AJ	0.0030	203x
244115	*CHRNE*	Myasthenic syndrome, congenital^	full AJ	0.0030	203x
18242	*FKTN*	Walker-Warburg syndrome*	full AJ<br>part AJ	0.0055<br>0.0046	186x<br>155x
15076	*FAM161A*	Retinitis pigmentosa	full AJ	0.0025	169x
32701	*BRCA1*	Breast-ovarian cancer, familial 1	full AJ	0.0030	102x
28743	*PEX2*	Zellweger syndrome*	full AJ	0.0045	102x
32049	*GJB2*	Nonsyndromic hearing loss and deafness	full AJ<br>part AJ	0.0189<br>0.0084	99x<br>44x
286483	*SLC3A1*	Cystinuria	full AJ<br>part AJ	0.0070<br>0.0046	95x<br>62x
274143	*EYS*	Retinitis pigmentosa	full AJ<br>part AJ	0.0055<br>0.0046	93x<br>78x
26930	*F11*	Hereditary factor XI deficiency disease*	full AJ<br>part AJ	0.0224<br>0.0099	76x<br>34x
94265	*CCDC65*	Primary ciliary dyskinesia	full AJ<br>part AJ	0.0070<br>0.0038	68x<br>37x
19972	*PCDH15*	Usher syndrome type 1*	part AJ<br>full AJ	0.0046<br>0.0040	62x<br>54x
22168	*CFTR*	Cystic fibrosis+	full AJ	0.0080	60x
24364	*BRCA2*	Breast-ovarian cancer, familial 2	full AJ<br>part AJ	0.0050<br>0.0038	56x<br>43x
18928	*HEXA*	Tay-Sachs disease+	part AJ<br>full AJ	0.0107<br>0.0105	36x<br>36x
415113	*NCF*	Chronic granulomatous disease^	full AJ<br>part AJ	0.0095<br>0.0046	32x<br>16x
20180	*USH1C*	Usher syndrome	full AJ	0.0040	14x

Table S4. The p-values for the 42 enriched and potentially deleterious variants identified in non-AJ UK individuals.

MAF_{REG}: regional MAF of the variant

MAF_{NFE}: MAF of the variant in Non-Finnish European individuals in gnomAD

These values are computed using one-sided Fisher's Exact Test (significance determined after Bonferroni correction for multiple testing).

ClinVar Variant Allele ID	Gene	Condition	Region	MAF_{REG}	Enrichment (vs MAF_{NFE})	p-value
33889	*CLCN1*	Congenital myotonia	Shetland	0.0061	138x	6.7E-10
21837	*ADGRV1*	Usher syndrome	Shetland	0.0061	104x	1.7E-09
			Strathclyde	0.0012	21x	6.5E-05
23042	*RDH5*	Fundus albipunctatus	Orkney	0.0088	25x	8.9E-10
23943	*PPT1*	Neuronal ceroid lipofuscinosis 1	Shetland	0.0122	17x	5.0E-11
21382	*FANCF*	Fanconi anaemia	Strathclyde	0.0017	8.8x	8.3E-05
20604	*AIPL1*	Leber congenital amaurosis	Shetland	0.0061	8.8x	1.1E-04
20006	*ABCG8*	Sitosterolaemia 1	Shetland	0.0102	8.8x	6.1E-07
16367	*BBS10*	Bardet-Biedl syndrome	Shetland	0.0071	6.6x	1.5E-04
			Orkney	0.0069	6.4x	1.9E-04
176561	*LOXHD1*	Nonsyndromic hearing loss and deafness	Scotland SE	0.0068	6.0x	3.9E-05
20826	*SLC7A9*	Cystinuria	Wales S	0.0039	44x	1.3E-21
133510	*CHEK2*	Hereditary cancer-predisposing syndrome	Wales N	0.0051	43x	8.4E-11
			Wales S	0.0026	22x	5.0E-13
71108	*NPHS1*	Finnish congenital nephrotic syndrome	Wales S	0.0025	42x	3.6E-14
16142	*AGL*	Glycogen Storage Disease Type III	Wales S	0.0009	32x	1.0E-05
815895	*SMARCAL1*	Schimke immuno-osseous dysplasia	Wales S	0.0012	28x	4.2E-07
203537	*GAMT*	Deficiency of guanidinoacetate methyltransferase	Wales S	0.0017	19x	1.6E-08
27983	*SPR*	Dystonia	Wales S	0.0019	16x	1.2E-08
105746	*CEP290*	Leber congenital amaurosis	Wales S	0.0014	14x	1.8E-06
414917	*MME*	Charcot-Marie-Tooth disease, axonal, type 2T	Wales S	0.0015	5.2x	1.4E-04
226048	*PEX6*	Zellweger syndrome	Lancs	0.0022	73x	6.0E-13
32480	*COL7A1*	Dystrophic epidermolysis bullosa	Notts	0.0010	65x	2.1E-08
			Lancs	0.0008	57x	3.5E-06
186978	*G6PC*	Glycogen storage disease	Yorks	0.0009	62x	1.1E-05
185684	*CLPB*	3-methylglutaconic aciduria with cataracts, neurologic involvement, and neutropenia	Notts	0.0008	57x	1.9E-07
359454	*COL7A1*	Dystrophic epidermolysis bullosa	Yorks	0.0008	52x	2.9E-05
431680	*PDE6A*	Retinitis pigmentosa	Staffs	0.0007	48x	7.3E-06
19010	*ALMS1*	Alstrom syndrome	Lancs	0.0020	27x	3.4E-10
			Staffs	0.0017	23x	1.8E-09
			Notts	0.0012	16x	4.5E-07
611902	*PEPD*	Prolidase deficiency	Notts	0.0010	22x	2.5E-06
249211	*FIG4*	Amyotrophic lateral sclerosis	Notts	0.0006	20x	2.8E-04
190033	*DRAM2*	Retinal Dystrophy	Lancs	0.0012	20x	5.8E-06
100244	*DYSF*	Limb-girdle muscular dystrophy	Staffs	0.0010	14x	3.3E-05
100251	*DYSF*	Limb-girdle muscular dystrophy	Staffs	0.0010	14x	3.3E-05
26935	*F11*	Hereditary factor XI deficiency disease	Lancs	0.0013	13x	7.2E-06
			Yorks	0.0011	10x	7.9E-05
33396	*CHRNE*	Myasthenic syndrome	Lancs	0.0017	13x	5.8E-07
240612	*DNAI1*	Primary ciliary dyskinesia	Staffs	0.0011	11x	2.1E-05
29030	*PNP*	Purine-nucleoside phosphorylase deficiency	England NE	0.0012	10x	7.9E-05
443176	*COL4A4*	Alport syndrome	Staffs	0.0014	9.6x	4.0E-06
			Lancs	0.0013	9.0x	3.9E-05
203474	*PNPO*	Pyridoxal phosphate-responsive seizures	Staffs	0.0017	9.6x	4.3E-07
16237	*MARVELD2*	Deafness, autosomal recessive 49	Lancs	0.0015	8.5x	1.8E-05
272434	*SLC7A9*	Cystinuria	Lancs	0.0022	8.2x	3.4E-07
489962	*TECTA*	Nonsyndromic hearing loss and deafness	Notts	0.0012	5.8x	1.1E-04
76544	*DNAH5*	Primary Ciliary Dyskinesia	Lancs	0.0020	5.2x	3.0E-05
401365	*SPG7*	Hereditary spastic paraplegia	Ireland	0.0020	9.0x	2.1E-05
31343	*FMO3*	Trimethylaminuria	Ireland	0.0040	5.5x	3.9E-07

Table S5. The enriched and potentially deleterious frameshift variant in the *OBSL1* gene (ClinVar Variant Allele ID: 192467, Three M syndrome 2).

MAF_{REG}: regional MAF of the variant

MAF_{NFE}: MAF of the variant in Non-Finnish European individuals in gnomAD

Region	MAF_{REG}	Enrichment vs MAF_{NFE}	Reason not classified as regionally enriched
Orkney	0.0079	38x	n/a
Shetland	0.0051	25x	
Wales N	0.0045	22x	
Wales S	0.0014	6.8x	
Scotland SE	0.0015	7.3x	found in <5 individuals
Hampshire&Wiltshire	0.0013	6.3x	fails Fisher's test
England NE	0.0010	4.9x	enrichment <5-fold
Notts	0.0007	3.5x	
Strathclyde	0.0007	3.5x	
Lancs	0.0007	3.2x	
Scotland NE	0.0006	2.9x	
Staffs	0.0004	2.1x	
Yorks	0.0003	1.5x	
Irish	0.0002	1.2x	
London	0.0002	0.8x	not enriched
full AJ, part AJ, East Anglia, England SW, Kent	not found		not found

Table S6. Estimate of variant frequency fluctuations observed in the 20 UKB regions w.r.t. Non-Finnish Europeans (NFE) in gnomADg 3.1.1. The numbers for each region represent the amount the SNPs in each category as a proportion of all regional SNPs found in NFE individuals with $1\% \leq MAF_{NFE} < 5\%$; rows sorted on total enriched values.

	depleted			no change	enriched		
	total	> 4 fold	2-4 fold		2-4 fold	> 4 fold	total
full AJ	32.00%	14.68%	17.32%	55.31%	11.19%	1.50%	12.69%
part AJ	10.90%	2.00%	8.90%	84.28%	4.65%	0.17%	4.82%
Shetland	15.26%	3.62%	11.64%	81.12%	3.53%	0.09%	3.62%
Orkney	13.74%	3.39%	10.35%	83.19%	2.98%	0.08%	3.06%
Irish	7.87%	1.72%	6.15%	90.94%	1.10%	0.08%	1.18%
Wales N	6.20%	1.40%	4.80%	93.03%	0.70%	0.07%	0.77%
Wales S	6.13%	1.39%	4.74%	93.39%	0.41%	0.07%	0.48%
Scotland SE	5.14%	1.29%	3.85%	94.44%	0.36%	0.07%	0.43%
Strathclyde	5.61%	1.40%	4.21%	93.98%	0.34%	0.07%	0.41%
Scotland NE	5.11%	1.32%	3.79%	94.51%	0.31%	0.06%	0.37%
Lancs	5.01%	1.27%	3.74%	94.65%	0.28%	0.07%	0.35%
Staffs	5.03%	1.26%	3.77%	94.64%	0.27%	0.07%	0.34%
England NE	4.38%	1.18%	3.20%	95.32%	0.23%	0.07%	0.30%
Notts	4.33%	1.24%	3.09%	95.39%	0.23%	0.05%	0.28%
Yorks	4.19%	1.10%	3.09%	95.55%	0.19%	0.07%	0.26%
Kent	4.12%	1.26%	2.86%	95.65%	0.16%	0.07%	0.23%
England SW	4.28%	1.15%	3.13%	95.52%	0.14%	0.07%	0.21%
Hamp&Wilt	4.10%	1.27%	2.83%	95.69%	0.13%	0.08%	0.21%
London	4.17%	1.18%	2.99%	95.62%	0.14%	0.06%	0.20%
East Anglia	4.05%	1.16%	2.89%	95.75%	0.13%	0.06%	0.19%

Table S7. Estimate of regional nucleotide diversity π based on known SNPs found with MAF > 5% in our 20 region dataset

region	π
full AJ	0.299322
Shetland	0.300563
Orkney	0.301201
Wales_N	0.301301
Wales_S	0.301424
Irish	0.301425
London	0.301534
Strath	0.301549
Scotland_NE	0.301550
Notts	0.301597
England_SW	0.301641
HampWilt	0.301677
Kent	0.301680
England_NE	0.301710
Yorks	0.301721
Scotland_SE	0.301776
Lancs	0.301811
Staffs	0.301846
East_Anglia	0.301906
part AJ	0.302192

Table S8. Estimate of regional strength of purifying selection based on variants with regional MAF ≤ 1% and not reported in the full gnomAD dataset

LOF: Loss of function variants; synon: synonymous variants

region	total LOF SNPs	total synon SNPs	mean LOF SNPs	mean synon SNPs	mean LOF/synon ratio
London	396	5,565	0.80	11.16	7.17%
England_NE	394	5,106	0.80	10.41	7.70%
Kent	434	5,603	0.88	11.30	7.76%
England_SW	447	5,455	0.90	11.09	8.14%
East_Anglia	473	5,802	0.96	11.80	8.17%
HampWilt	447	5,407	0.91	10.89	8.32%
Staffs	408	4,874	0.85	10.15	8.40%
Wales_N	382	4,525	0.79	9.33	8.52%
Yorks	444	5,252	0.91	10.65	8.59%
Irish	339	3,987	0.70	8.09	8.61%
Lancs	408	4,544	0.83	9.31	8.96%
Scotland_SE	392	4,318	0.80	8.89	9.05%
Orkney	304	3,093	0.87	9.61	9.06%
Wales_S	434	4,766	0.89	9.75	9.11%
Scotland_NE	415	4,574	0.86	9.38	9.15%
Shetland	285	2,917	0.95	10.36	9.17%
Notts	501	5,507	1.04	11.25	9.21%
Strath	373	3,929	0.76	8.02	9.51%
part AJ	339	3,429	0.68	6.93	9.85%
full AJ	173	1,570	0.35	3.31	10.64%

Table S9. Variant QC filtering statistics, reporting the number of sites filtered at each step (as a proportion of the sites submitted to it)

	Filtering based on					Filtering based on DP, GQ and VAF			
**Region**	**miss≥10%**	**not in target regions**	**gnomAD FAIL**	**in LCR**	**SNPs in PCW**	**individual SNP calls**	**individual INDEL calls**	**SNP sites**	**INDEL sites**
Shetland	4.35%	54.99%	3.94%	1.37%	0.48%	0.78%	4.51%	0.50%	1.92%
Orkney	4.16%	54.89%	3.85%	1.38%	0.48%	0.78%	4.59%	0.47%	1.77%
Scotland SE	3.38%	54.06%	3.30%	1.29%	0.42%	0.70%	4.00%	0.74%	2.85%
Scotland NE	2.58%	52.87%	2.73%	1.16%	0.39%	0.71%	4.17%	0.70%	2.82%
Strathclyde	2.38%	52.62%	2.60%	1.15%	0.39%	0.72%	4.10%	0.68%	2.97%
Wales N	3.33%	53.75%	3.19%	1.26%	0.42%	0.73%	4.37%	0.77%	2.98%
Wales S	2.04%	52.10%	2.35%	1.06%	0.37%	0.70%	4.10%	0.67%	2.66%
Irish	2.63%	52.76%	2.72%	1.18%	0.39%	0.74%	4.37%	0.77%	3.22%
East Anglia	3.05%	53.47%	3.01%	1.18%	0.39%	0.75%	4.39%	0.74%	3.20%
England NE	1.97%	52.14%	2.31%	1.07%	0.35%	0.71%	4.13%	0.66%	2.77%
England SW	2.61%	52.88%	2.71%	1.16%	0.39%	0.73%	4.25%	0.69%	3.00%
Hamp&Wilt	2.28%	52.48%	2.49%	1.13%	0.37%	0.72%	4.25%	0.71%	3.03%
Kent	2.68%	52.98%	2.72%	1.15%	0.38%	0.75%	4.26%	0.71%	3.29%
Lancs	2.12%	52.20%	2.40%	1.08%	0.36%	0.75%	4.39%	0.70%	2.87%
Yorks	1.88%	51.98%	2.24%	1.06%	0.35%	0.71%	4.18%	0.63%	2.66%
Notts	1.77%	51.78%	2.13%	1.01%	0.34%	0.73%	4.30%	0.66%	2.79%
Staffs	2.00%	52.20%	2.35%	1.07%	0.38%	0.71%	4.15%	0.65%	2.92%
London	1.37%	51.05%	1.77%	0.89%	0.29%	0.75%	4.41%	0.58%	2.26%
all AJ	3.08%	54.03%	3.06%	1.21%	0.45%	0.75%	4.46%	0.78%	3.31%

miss≥10%: sites excluded due to more than 10% of the individuals are with missing genotype

not in target regions: sites excluded due to variant location being outside of the WES capture target region

gnomAD FAIL: sites excluded due to being found to fail the gnomADg QC

in LCR: sites excluded due to variant location in Low-Complexity Region (LCR)

SNPs in PCW: SNP sites excluded due to being in Poor Coverage Window (PCW) region in gnomADg (defined as 10bp windows centred on any base with coverage < 10x)

individual SNP calls: individual SNP genotypes being reset to REF due to poor DP, GQ and/or VAF; leading to excluding the corresponding number of **SNP sites**

individual INDEL calls: individual INDEL genotypes being reset to REF due to poor DP, GQ and/or VAF; leading to excluding the corresponding number of **INDEL sites**
