# Supplementary material for: Regionally enriched rare deleterious exonic variants in the UK and Ireland
Source: Nat Commun. 2024 Oct 2;15:8454. doi: 10.1038/s41467-024-51604-2 (PMC11446911; doi:10.1038/s41467-024-51604-2)
Supplement: Supplementary file 2 — Reporting Summary [file 41467_2024_51604_MOESM2_ESM.pdf]

nature portfolio

Corresponding author(s): Mihail Halachev

Last updated by author(s): Jul 30, 2024

Reporting Summary

Nature Portfolio wishes to improve the reproducibility of the work that we publish. This form provides structure for consistency and transparency in reporting. For further information on Nature Portfolio policies, see our Editorial Policies and the Editorial Policy Checklist.

Statistics

For all statistical analyses, confirm that the following items are present in the figure legend, table legend, main text, or Methods section.

n/a	Confirmed	
☐	☒	The exact sample size (*n*) for each experimental group/condition, given as a discrete number and unit of measurement
☐	☒	A statement on whether measurements were taken from distinct samples or whether the same sample was measured repeatedly
☐	☒	The statistical test(s) used AND whether they are one- or two-sided<br>*Only common tests should be described solely by name; describe more complex techniques in the Methods section.*
☐	☒	A description of all covariates tested
☐	☒	A description of any assumptions or corrections, such as tests of normality and adjustment for multiple comparisons
☐	☒	A full description of the statistical parameters including central tendency (e.g. means) or other basic estimates (e.g. regression coefficient) AND variation (e.g. standard deviation) or associated estimates of uncertainty (e.g. confidence intervals)
☐	☒	For null hypothesis testing, the test statistic (e.g. *F*, *t*, *r*) with confidence intervals, effect sizes, degrees of freedom and *P* value noted<br>*Give P values as exact values whenever suitable.*
☒	☐	For Bayesian analysis, information on the choice of priors and Markov chain Monte Carlo settings
☒	☐	For hierarchical and complex designs, identification of the appropriate level for tests and full reporting of outcomes
☒	☐	Estimates of effect sizes (e.g. Cohen's *d*, Pearson's *r*), indicating how they were calculated

Our web collection on statistics for biologists contains articles on many of the points above.

Software and code

Policy information about availability of computer code

Data collection	No software was used for data collection.
Data analysis	All software used in this work is publicly available. It is described (with versions) throughout the main text of the manuscript and repeated here for convenience.<br><br>The WES sequencing, read mapping and variant calling for the ~200k UKBB participants and for the individuals from the Northern Isles (Shetland and Orkney) was performed by Regeneron following the OQFE protocol. The starting point for our analyses were the project VCF files generated by the OQFE protocol.<br><br>Publicly available software and datasets used in this study (in alphabetical order)<br>BCFtools v1.10.2<br>gnomADg v3.1.1<br>PHYLIP v3.697<br>PLINK v1.90b4<br>PRIMUS v1.9.0<br>R package qgraph v 1.9.8<br>VEP v102<br>VCFtools v0.1.13

For manuscripts utilizing custom algorithms or software that are central to the research but not yet described in published literature, software must be made available to editors and reviewers. We strongly encourage code deposition in a community repository (e.g. GitHub). See the Nature Portfolio guidelines for submitting code & software for further information.

Data

Policy information about availability of data

All manuscripts must include a data availability statement. This statement should provide the following information, where applicable:

- Accession codes, unique identifiers, or web links for publicly available datasets
- A description of any restrictions on data availability
- For clinical datasets or third party data, please ensure that the statement adheres to our policy

For ORCADES and VIKING the research data and/or DNA samples are available through managed access by application (accessQTL@ed.ac.uk), following approval by the QTL Data Access Committee with expected timeframe for response of about 2 months. These data are available under managed access due to the consent given by the participants and Research Ethics Committee approvals. Each approved project is subject to a data or materials transfer agreement (D/MTA) or commercial contract. Data may be shared with academic or commercial recipients worldwide and may be used within the parameters of the Research Ethics Committee approvals. The UK Biobank genotypic data used in this study were approved under application 19655 and are available to qualified researchers via the UK Biobank managed data access process. All other data supporting the findings described in this manuscript (e.g., gnomAD, ClinVar, CADD scores, etc) are publicly available.

Research involving human participants, their data, or biological material

Policy information about studies with human participants or human data. See also policy information about sex, gender (identity/presentation), and sexual orientation and race, ethnicity and racism.

Reporting on sex and gender	Because autosomal alleles assort independently of the sex chromosomes, there is no expectation of differences in allele frequencies between males and females; moreover, as dividing the samples in two would decrease the power of study and precision of estimates, no sex-based analyses were performed.
Reporting on race, ethnicity, or other socially relevant groupings	In Viking Genes, participants were included in the study if they had all four grandparents born in Orkney or all four in Shetland; hence there are no ethnic minority participants. From these sets, maximally unrelated subsets were selected for further analysis. In UK Biobank, we selected only participants who self-identify as “White British”, exhibit very similar genetic ancestry based on a principal component analysis of the UKB genome-wide SNP array genotypes (UKB field: 22006) and who were born outside large metropolitan areas in the corresponding region. The Irish participants had to self-identify as “irish” (UKB field: 21000) and be born in either Northern Ireland or the Republic of Ireland (UKB field: 1647). The participants satisfying the above criteria for each region were then evaluated for relatedness and the maximum unrelated set per region generated as for the Northern isles cohorts.
Population characteristics	The populations analysed were not chosen on the basis of age or any phenotypic characteristic or diagnosis. They were rather unrelated healthy adult volunteer members of the respective cohorts, representing different geographic ancestries across the British Isles and Ireland. All were subject to whole exome sequencing and sub-populations were chosen according to the birthplaces of participants (for UK Biobank) or grandparents of participants (for Viking Genes).
Recruitment	UK Biobank recruitment is detailed in Sudlow et al (2015) PLOS Medicine. DOI:10.1371/journal.pmed.1001719. Recruitment in Viking Genesis described in Kerr et al (2019) Scientific Reports 9:10964.
Ethics oversight	All participants in the Viking Health Study - Shetland (VIKING) gave written informed consent for broad ranging health and ancestry research including whole genome/exome sequencing and the study was given a favourable opinion by the South East Scotland Research Ethics Committee (REC Ref 12/SS/0151). All participants in the Orkney Complex Disease Study (ORCADES) gave written informed consent for broad ranging health and population research, including sequencing and the study was approved by Research Ethics Committees in Orkney, Aberdeen (North of Scotland REC) and South East Scotland REC, NHS Lothian (reference: 12/SS/0151). Ethics approval for the UK Biobank study was obtained from the North West Centre for Research Ethics Committee (11/NW/0382), and all participants gave written informed consent.

Note that full information on the approval of the study protocol must also be provided in the manuscript.

Field-specific reporting

Please select the one below that is the best fit for your research. If you are not sure, read the appropriate sections before making your selection.

☒ Life sciences ☐ Behavioural & social sciences ☐ Ecological, evolutionary & environmental sciences

For a reference copy of the document with all sections, see nature.com/documents/nr-reporting-summary-flat.pdf

Life sciences study design

All studies must disclose on these points even when the disclosure is negative.

Sample size	No power calculation was performed. In population genetics the larger the sample size, the greater the ability to investigate rarer alleles, for there are more observations of each allele. The sample sizes here were constrained by the number of individuals born in each region and available in the 200k release of UK Biobank exomes. For analyses influenced by sample size, we set the sample sizes for each region to match the sizes available in the Orkney and Shetland Islands (both n~500), which was the maximum number of unrelated individuals available in those cohorts, leading to a total sample size of 10,001). For analyses less influenced by sample size we used the full dataset (n=44,696).

Data exclusions | Individuals from Viking Genes who did not have all four grandparents born in either Orkney or Shetland were excluded. In UK Biobank (UKB) we excluded individuals who were born outside of our 20 geographic regions or who were born within predefined urban areas, using circles of suitable radii to exclude large cities such as Glasgow, Newcastle, Liverpool, Manchester, Birmingham and Cardiff. This focused interest on people born outside large metropolitan areas where there has been increased movement over the last generations. UKB participants who withdrew their consent are excluded.

Replication | Without the recruitment of new cohorts with ~45,000 individuals from the regions of interest, it is not possible to replicate our findings. Such a dataset does not exist at present. We note that the structure which we observe in coding data reflects that seen in genome-wide chip data in earlier work.

Randomization | The present study does not describe a randomized control trial or an intervention study. There are no treatments or other interventions. The study is purely descriptive. Hence, allocation and randomisation are not relevant.

Blinding | The present study does not describe a blinded randomised controlled trial, thus it was not necessary to blind researchers. Subjects analysed in this study were volunteers from the general population. Allocation, treatment and randomisation are not relevant to this study.

Reporting for specific materials, systems and methods

We require information from authors about some types of materials, experimental systems and methods used in many studies. Here, indicate whether each material, system or method listed is relevant to your study. If you are not sure if a list item applies to your research, read the appropriate section before selecting a response.

Materials & experimental systems

n/a	Involved in the study
☒	☐ Antibodies
☒	☐ Eukaryotic cell lines
☒	☐ Palaeontology and archaeology
☒	☐ Animals and other organisms
☒	☐ Clinical data
☒	☐ Dual use research of concern
☒	☐ Plants

Methods

n/a	Involved in the study
☒	☐ ChIP-seq
☒	☐ Flow cytometry
☒	☐ MRI-based neuroimaging

Plants

Seed stocks | *Report on the source of all seed stocks or other plant material used. If applicable, state the seed stock centre and catalogue number. If plant specimens were collected from the field, describe the collection location, date and sampling procedures.*

Novel plant genotypes | *Describe the methods by which all novel plant genotypes were produced. This includes those generated by transgenic approaches, gene editing, chemical/radiation-based mutagenesis and hybridization. For transgenic lines, describe the transformation method, the number of independent lines analyzed and the generation upon which experiments were performed. For gene-edited lines, describe the editor used, the endogenous sequence targeted for editing, the targeting guide RNA sequence (if applicable) and how the editor was applied.*

Authentication | *Describe any authentication procedures for each seed stock used or novel genotype generated. Describe any experiments used to assess the effect of a mutation and, where applicable, how potential secondary effects (e.g. second site T-DNA insertions, mosiacism, off-target gene editing) were examined.*
